# Structural basis of metabolite transport by the chloroplast outer envelope channel OEP21

**DOI:** 10.1038/s41594-023-00984-y

**Published:** 2023-05-08

**Authors:** Umut Günsel, Kai Klöpfer, Elisabeth Häusler, Manuel Hitzenberger, Bettina Bölter, Laura E. Sperl, Martin Zacharias, Jürgen Soll, Franz Hagn

**Affiliations:** 1 Bavarian NMR Center (BNMRZ), Department of Bioscience, School of Natural Sciences, Technical University of Munich, Garching, Germany; 2 Institute of Structural Biology, Helmholtz Munich, Neuherberg, Germany; 3 Lehrstuhl für Theoretische Biophysik (T38), Department of Bioscience, School of Natural Sciences, Technical University of Munich, Garching, Germany; 4 Biozentrum, LMU München, Department of Biology, Planegg-Martinsried, Germany

**Keywords:** Solution-state NMR, Plant sciences

## Abstract

Triose phosphates (TPs) are the primary products of photosynthetic CO_2_ fixation in chloroplasts, which need to be exported into the cytosol across the chloroplast inner envelope (IE) and outer envelope (OE) membranes to sustain plant growth. While transport across the IE is well understood, the mode of action of the transporters in the OE remains unclear. Here we present the high-resolution nuclear magnetic resonance (NMR) structure of the outer envelope protein 21 (OEP21) from garden pea, the main exit pore for TPs in C_3_ plants. OEP21 is a cone-shaped β-barrel pore with a highly positively charged interior that enables binding and translocation of negatively charged metabolites in a competitive manner, up to a size of ~1 kDa. ATP stabilizes the channel and keeps it in an open state. Despite the broad substrate selectivity of OEP21, these results suggest that control of metabolite transport across the OE might be possible.

## Abstract (web summary)

Günsel et al. unravel the structural and functional properties of OEP21, a major metabolite channel in the chloroplast outer envelope, and show that this porin utilizes a promiscuous mechanism to control the passage of a distinct set of substrates.

## Main

**Fig. 1:**
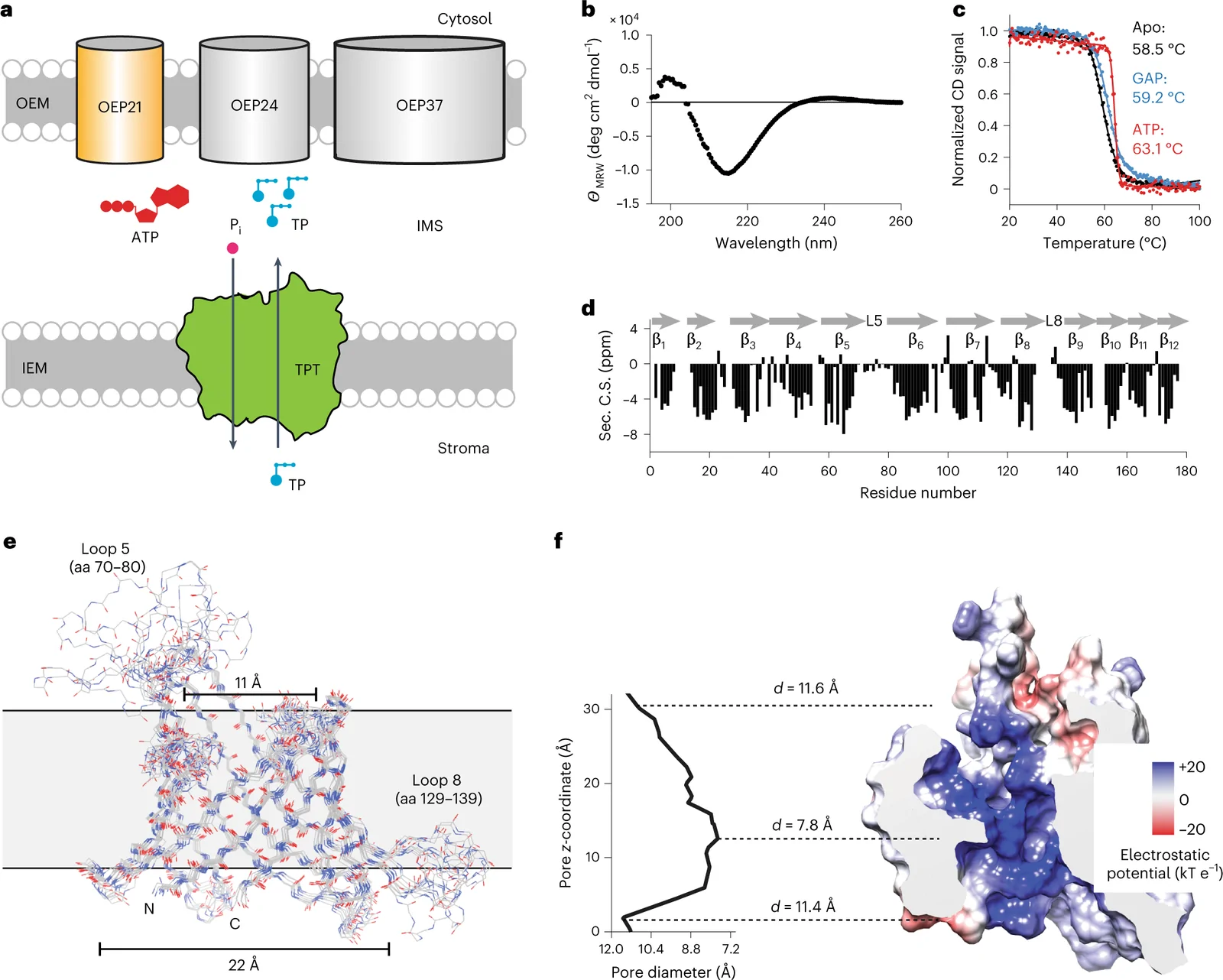
OEP21 is a highly positively charged β-barrel porin in the OE membrane. **a**, TP transport across the IE and OE membranes by the TP/P_i_ translocator (TPT, PDB:5Y78)^40^ and OE proteins (OEPs) of various sizes, such as OEP21, OEP24 and OEP37. P_i_, inorganic phosphate. **b**, Far-UV CD spectrum of recombinant OEP21 in LDAO micelles. *Θ*_MRW_, ellipticity mean residue weight. **c**, Thermal stability of apo-OEP21 and in presence of 0.5 mM GAP or ATP. **d**, NMR secondary chemical shift (Sec. C.S.) information indicating the presence of 12 β-strand regions of OEP21. **e**, NMR structural bundle of the OEP21 β-barrel pore showing well-defined secondary structure elements (r.m.s.d. of 0.5 Å) and a funnel-like shape. **f**, Analysis of the pore geometry, indicating a 7.8-Å-wide constriction site on the electrostatic potential surface map of the pore interior (blue indicates positively charged regions, and negatively charged regions are shown in red). Source data

Chloroplasts are the primary sites of photosynthesis in plant cells. During photosynthesis, ATP and NADPH are generated and used for fixation of CO_2_ into the TP d-glyceraldehyde-3-phosphate (GAP) through the Calvin–Benson–Bassham cycle in the chloroplast stroma. These primary products must be transported across the IE and OE membranes into the cytosol for further metabolic processing and energy supply^1^. Each of these membranes is equipped with a distinct set of ion channels and transporters that enable transport of nutrients, solutes and metabolites (Fig. 1a). Transport across the IE membrane is mediated by a diverse set of proteins (for example, TGD-1, PPT, TPT, NNT)^2^, whereas the OE membrane was initially considered to be a permeable sieve that cannot form a barrier for small molecules^3^. This view was questioned by the finding that the OE contains multiple substrate and charge-selective channels^4^, suggesting a more specific transport mechanism^5^. Beside TOC75 (translocase of the outer chloroplast membrane of 75 kDa), which is required for pre-protein import into chloroplasts, more channels for the transport of metabolites and solutes have been discovered^6,7^. This includes the amine and amino acid channel OEP16 (ref. ^8^), OEP21 (ref. ^9^) and the cation-selective channels OEP23 (ref. ^10^), OEP24 (ref. ^11^) and OEP37 (ref. ^12^). TP translocation across the OE membrane is mainly conducted by OEP21 and OEP24 (refs. ^6,7,13^) (Fig. 1a). To account for the different levels of metabolite flux in distinct plant species, the abundance of individual OEPs can vary strongly. The larger and less selective OEP24 pore is abundant in C_4_ plants, in which a higher rate of carbon fixation and metabolite flux occurs^13^. By contrast, the outward-rectifying OEP21 channel is more prominent in C_3_ plants and has been shown to interact with ATP, TPs and phosphate in a competitive manner^9,14^. However, the molecular and structural features of metabolite transport and selectivity remain elusive for OEP21, as well as the entire OEP channel family.

Here, we used NMR spectroscopy to determine the high-resolution structure of OEP21 from garden pea. We show that this channel consists of 12 β-strands that form a cone-shaped β-barrel pore, with the wider opening oriented toward the chloroplast intermembrane space (IMS). The inside of the pore is highly positively charged, suggesting specific binding and translocation of negatively charged metabolites. Metabolites interact with OEP21 in a charge-dependent and competitive manner. Interestingly, binding of ATP stabilizes the channel and affects the oligomer-to-monomer equilibrium. Using NMR and molecular dynamics (MD) simulations, we show that the translocation trajectory of GAP is guided by patches of positive charges in the channel. Finally, we show that not only TPs, but also larger molecules up to a molecular weight of ~1 kDa can pass OEP21. Taken together, these data provide detailed mechanistic insights into the functionality of an important member of the OEP family and suggest that these pores show a distinct level of selectivity and might be able to respond to changes in the cellular milieu in plants.

## Results

### OEP21 is a β-barrel membrane pore with a highly positively charged interior surface

**Extended Data Fig. 1:**
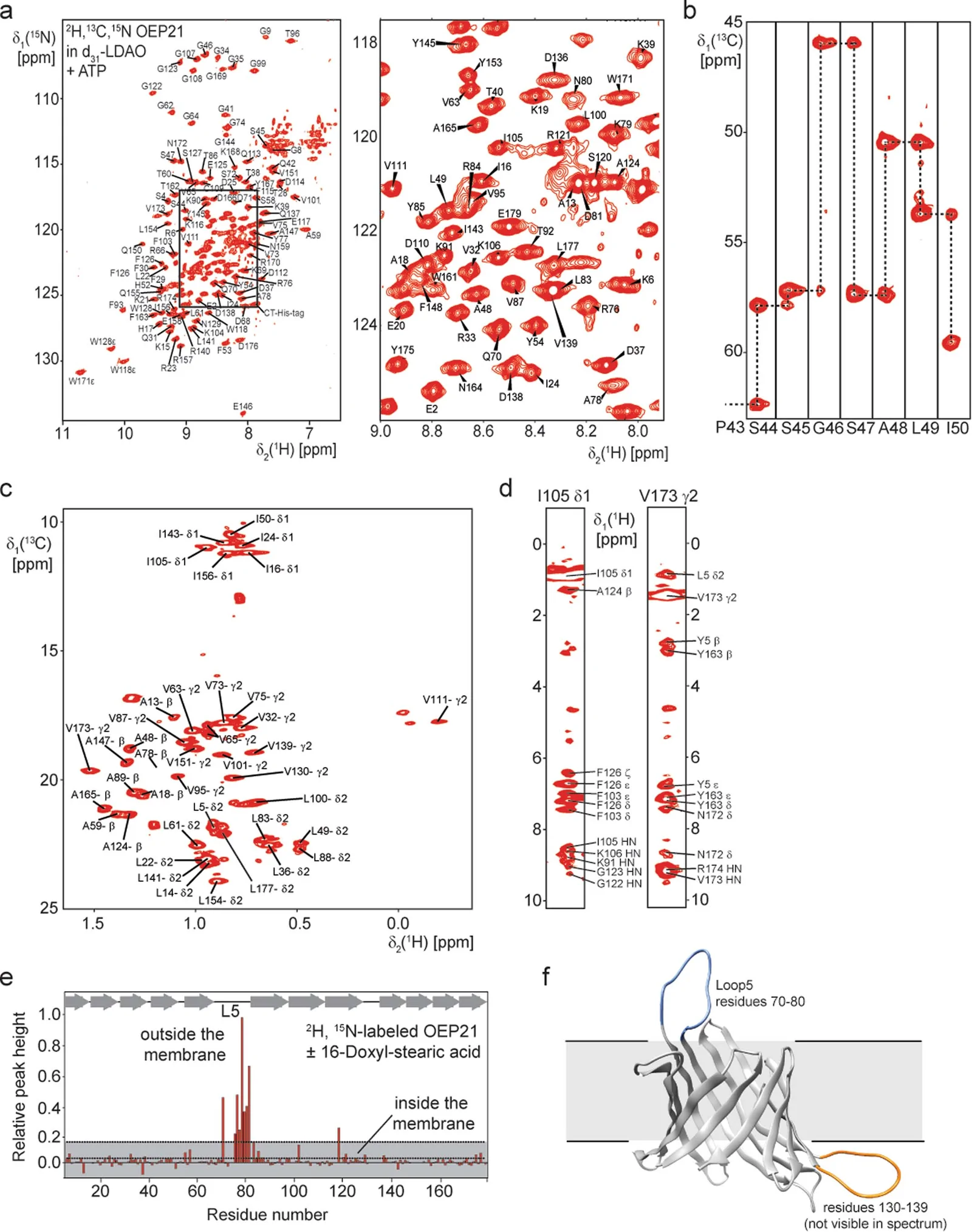
NMR assignments of OEP21 in LDAO micelles and its membrane location. 3D-triple resonance NMR experiments were performed with a 400 µM ^2^H,^13^C,^15^N-labeled OEP21 in 20 mM Na-phosphate pH 6.0, 50 mM NaCl, 0.5 mM EDTA, 5 mM DTT, 5 mM ATP, 300 mM *d*_*31*_-LDAO and 5 % D_2_O at 308 K. **(a)** All assigned NMR resonances in the 2D-[^15^N,^1^H]-TROSY are labeled. right panel: expansion of the central crowded spectral region marked by a box in the left panel. **(b)** Strips of a 3D-tr-HNCA NMR experiment at 308 K with ^2^H,^13^C,^15^N-labeled OEP21 in *d*_*31*_-LDAO micelles in complex with ATP run in a non-uniformly sampled (NUS) manner shows the sequential contacts required for backbone resonance assignment. **(c)** Assignments of (stereo-) specifically labeled methyl side-chains in OEP21, as indicated by the labels in the shown 2D-[^13^C,^1^H]-HMQC spectrum. **(d)** Representative strips of a NUS 3D-^13^C-edited-[^1^H,^1^H]-NOESY-HMQC experiment with a (stereo-) specifically ILVA ^1^H,^13^C methyl-labeled and otherwise ^2^H,^12^C,^15^N-labeled OEP21 sample with incorporated ^1^H,^15^N-labeled Phe and Tyr residues in *d*_*31*_-LDAO micelles. **(e)** Relative peak heights of OEP21 resonances derived from 2D-[^15^N,^1^H]-TROSY experiments in 150 mM LDAO or LDAO micelles supplemented with 2 mM 16-doxyl-stearic acid (16-DSA), corresponding to one 16-DSA molecule per micelle. **(f)** Resonances that are still visible in the 16-DSA sample are mainly located in L5 outside the membrane. Resonances of residues located in the membrane are broadened beyond detection due to the paramagentic effect of the free radical.

We first optimized protein refolding conditions for OEP21 in detergent micelles and identified the detergent lauryldimethylamine-*N*-oxide (LDAO), which has been previously reported to be suitable for the refolding of β-barrel membrane proteins^15,16^. An analysis of OEP21 in LDAO micelles by far-ultraviolet (UV) circular dichroism (CD) spectroscopy indicated that the protein had β-sheet secondary structure (Fig. 1b). Because OEP21 has been reported to bind or transport GAP and ATP^9^, we next performed CD-detected thermal melting experiments (Fig. 1c) in the presence of these molecules. GAP, and especially ATP, led to stabilization of OEP21. ATP is present at a concentration of ~1–2 mM in plant cells^17^. Thus, we added ATP to the OEP21 sample for the subsequent NMR structure determination. With this setup, we obtained high-quality two-dimensional (2D) and multidimensional NMR spectra (Extended Data Fig. 1a,b), which enabled us to obtain sequence-specific backbone resonance assignments. An analysis of the secondary ^13^C chemical shift information indicated that there were 12 β-strand regions of varying lengths (Fig. 1d), in contrast to the primary-sequence-based predicted secondary structure content (8 β-strands)^9^, but in good agreement with the prediction of the program AlphaFold^18^. High-resolution NMR structure determination was conducted with a uniformly ^2^H- and ^15^N-labeled and selectively methyl-group-labeled (Ile-δ1, Leu-δ2, Val-γ2, Ala-β) OEP21 sample bound to ATP for the acquisition of a set of heteronuclear three-dimensional (3D)-NOESY NMR experiments^19^. These data were used to assign the side chain methyl resonances (Extended Data Fig. 1c) and extract NOE distance restraints for structure calculation^20^ (Table 1 and Extended Data Fig. 1d), which resulted in a well-defined structural bundle of OEP21 showing a root mean square deviation (r.m.s.d.) of 0.5 Å in ordered secondary structure elements (Fig. 1e and Table 1). The overall shape of OEP21 resembles a funnel with a diameter of 22 Å on one side and 11 Å on the opposite side. Outside the membrane, most connecting loops are structurally well-defined, with a length of 3–7 amino acids (aa), except for the two longer loops (L5: aa 70–80 and L8: aa 129–139) that are located on each side of the β-barrel (Fig. 1e). L8 is not visible in the NMR spectra to a large extent, presumably owing to µs-to-ms motions. By contrast, L5 gives rise to strong NMR signals but does not show NOE contacts to other parts of the protein, indicating intrinsic flexibility. Solvent accessibility of L5 was probed by NMR paramagnetic relaxation enhancement (PRE) experiments using a spin-labeled fatty acid (12-doxyl-stearic acid) that led to signal attenuation of membrane-incorporated parts of OEP21 (Extended Data Fig. 1e,f), but left L5 unaffected. An analysis of the inner pore diameter (Fig. 1f) indicates that the most constricted position of the channel is 7.8 Å wide. The dimensions of the OEP21 pore are in good agreement with previously published electrophysiology data^14^, which led to estimations of a wider vestibule of 2.4 nm and a restriction zone of ~1 nm. The determined structure reveals that the pore has a highly positively charged inner surface (Fig. 1f), which suggests that it binds to metabolites in a charge-dependent manner, supported by the increased thermal stability when bound to GAP and ATP (Fig. 1c).

**Table 1:** NMR and refinement statistics for ATP-bound OEP21 in LDAO micelles

	OEP21
**NMR distance and dihedral constraints**	
Distance constraints	
Total NOE	623
Intra-residue	106
Inter-residue	517
Sequential (|*i* – *j*| = 1)	154
Medium-range (|*i* – *j*| < 4)	57
Long-range (|*i* – *j*| > 5)	306
Intermolecular	0
Hydrogen bonds	110
Total dihedral angle restraints	255
ϕ	128
ψ	127
**Structure statistics**	
Violations (mean and s.d.)	
Distance constraints (Å)	0.035 ± 0.002
Dihedral angle constraints (°)	0.11 ± 0.04
Max. dihedral angle violation (°)	1.211
Max. distance constraint violation (Å)	0.203
Deviations from idealized geometry	
Bond lengths (Å)	0.00384 ± 0.00008
Bond angles (°)	0.87 ± 0.01
Impropers (°)	2.4 ± 0.1
Average pairwise r.m.s. deviation^a^ (Å)	
Heavy	1.1 ± 0.1
Backbone	0.5 ± 0.1

^a^Pairwise r.m.s. deviation was calculated among 10 refined structures within ordered secondary structure elements (residues 1–9, 14–25, 28–36, 44–54, 58–68, 81–94, 102–113, 117–128, 140–146, 151–158, 161–166, 169–176).

### Orientation and oligomeric state of OEP21 in the chloroplast OE

**Fig. 2:**
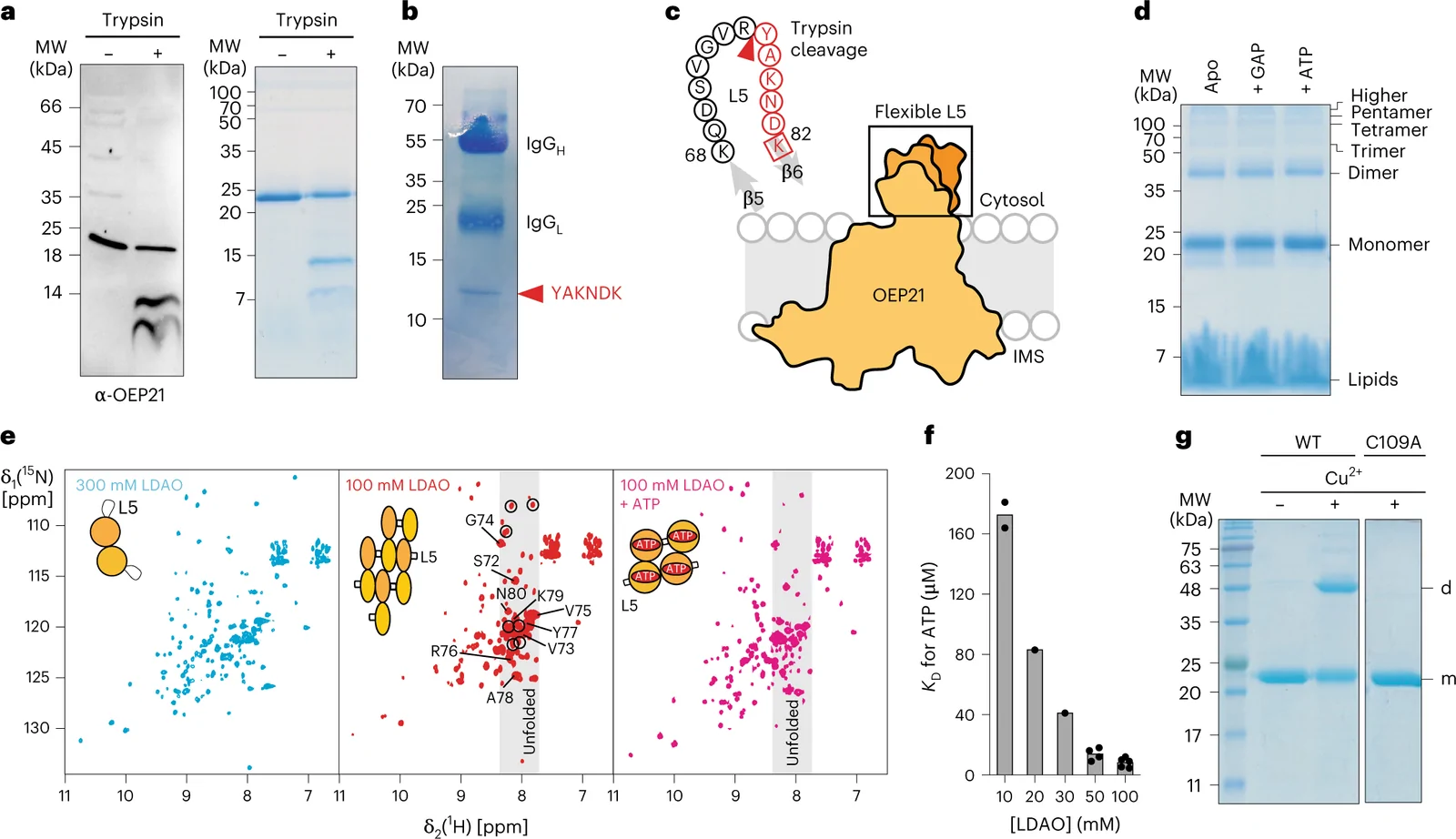
Oligomeric state and orientation of OEP21 in the chloroplast outer envelope. **a**, Analysis of trypsin-treated (+) or untreated (–) isolated OE vesicles (left) or recombinant OEP21 reconstituted liposomes (right) by immunoblotting against OEP21 or Coomassie staining. **b**, Coomassie-stained PVDF membrane from the immunoprecipitated trypsin-treated OEP21 fragment used for Edman sequencing. The immunoprecipitated fragment is indicated by the one-letter amino acid code, as are the positions of the light and heavy chains from the antiserum. **c**, Topology of OEP21 in the OE. The position of the trypsin cleavage site suggests that L5 is oriented toward the cytosol. **d**, Analysis of the oligomerization state of OEP21 in liposomes (20 μM) in the apo form or bound to 5 mM GAP or ATP by BS^3^ crosslinking. **e**, 2D-[^15^N,^1^H]-TROSY NMR experiments with ^2^H,^15^N-labeled OEP21 at high (light blue) and low (red) detergent conditions and in the presence of ATP (magenta). Encircled NMR signals in the random coil region are visible only in the oligomeric apo state. **f**, Affinity between OEP21 and ATP at the indicated LDAO concentrations, derived from ITC experiments. Bars represent the mean value of *n* ≥ 1 individual measurements. **g**, Non-reducing SDS–PAGE of WT OEP21 and OEP21-C109A (20 μM) in the absence (–) or presence (+) of oxidizing 1 mM Cu^2+^. d, dimer; m, monomer. **a**, **d** and **g** are representative of *n* ≥ 2 independent experiments. Source data

**Extended Data Fig. 2:**
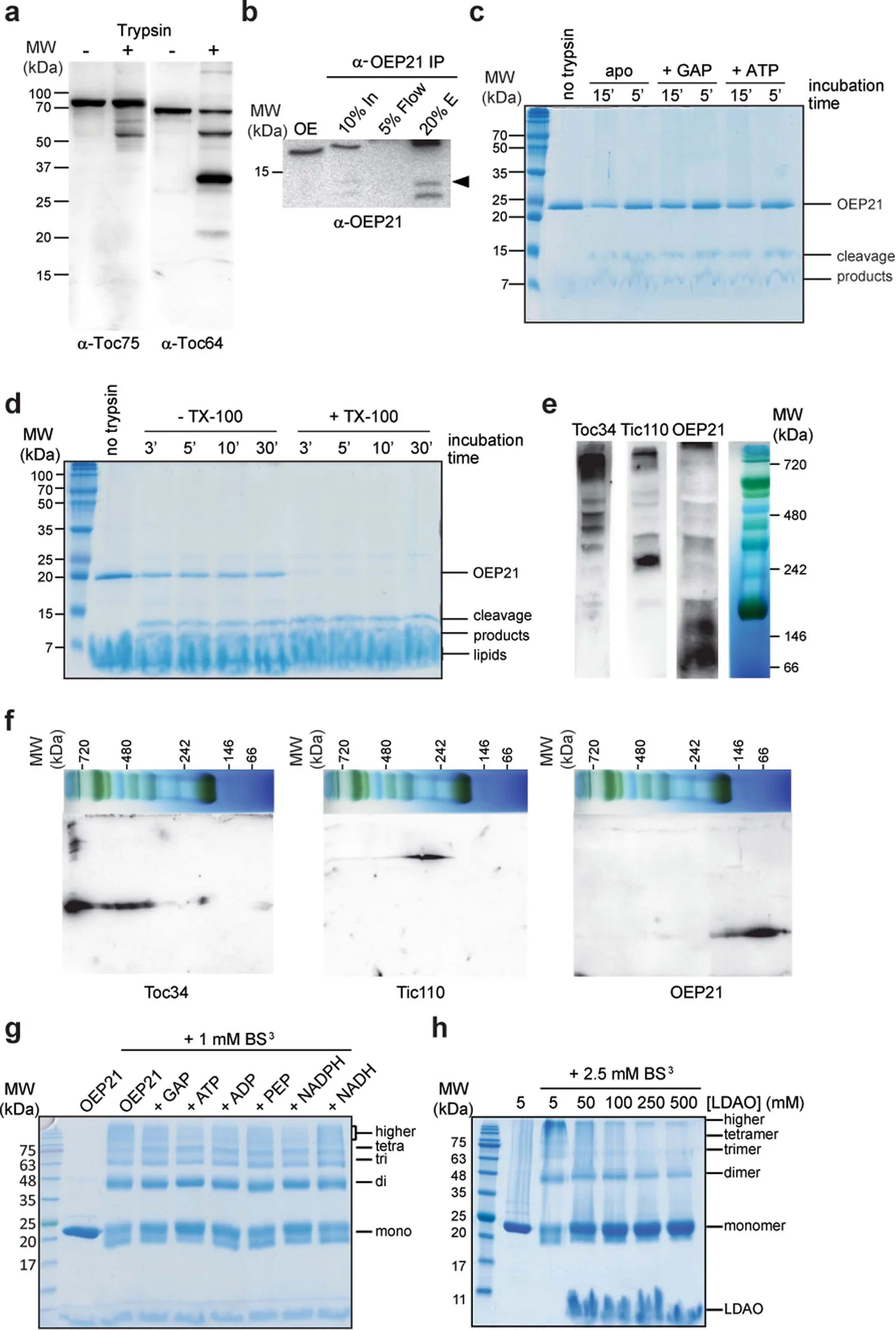
Orientation and oligomeric state of OEP21 in the OE membrane and reconstituted liposomes. **(a)** Isolated OE vesicles were treated (+) or not treated (-) with trypsin and analyzed by immunoblotting against Toc64 and Toc75. **(b)** Trypsin-treated OE was immunoprecipitated with antiserum against OEP21. 10% of the input (In), 5% flow through (Flow) and 20% elution (E) were analyzed by immunoblotting against OEP21. Arrowhead indicates the upper 14 kDa proteolytic fragment. **(c)** Trypsin digestion experiments with 10 µM recombinant OEP21 reconstituted in liposomes prepared with soy polar lipids in the apo form or in presence of 5 mM GAP or 5 mM ATP monitored by Coomassie-stained SDS-PAGE without or with 0.1 µM trypsin. **(d)** The orientation of 10 µM OEP21 in liposomes was analyzed by trypsin cleavage (0.4 µM) in the absence or presence of 0.2 % triton-100 (TX-100). **(e)** Chloroplasts enriched with OEV were solubilized with 1% DDM and separated by Blue Native PAGE in a first **(e)** and SDS PAGE in a second dimension **(f)** followed by immunoblotting with the indicated antibodies. Molecular weights are indicated to the right of the first-dimension gel slice as deduced by known complex sizes^65^. Toc34 and Tic110 were applied as controls for known complexes in the outer and inner envelope, respectively. **(g)** Detection of the oligomeric state of OEP21 (20 μM) in LDAO micelles in presence of metabolites (5 mM) using BS^3^ crosslinking. **(h)** BS^3^ crosslinking of OEP21 at increasing detergent concentrations from 5 to 500 mM. Figures (a), (c), (d), (e), (f), (g) and (h) are representatives of n≥2 independent experiments and (b) shows the blot from the sample used for Edmann sequencing. Source data

The funnel-like shape of OEP21 is in agreement with its proposed outward-rectifying properties^14^. Because metabolite flow is mainly directed from the IMS into the cytosol under light conditions of photosynthesis, an orientation with the wider opening in the IMS is plausible. To probe the orientation of OEP21 in the OE, we next applied limited proteolysis experiments with isolated right-side-out OE vesicles (OEVs)^21,22^. In agreement with published data^23^, treatment of OEVs with trypsin resulted in two specific fragments (Fig. 2a, left). Decoration of the same samples with antibodies against Toc64 and Toc75 (refs. ^24,25^) confirmed the efficiency of the trypsin treatment (Extended Data Fig. 2a), i.e. the large cytosolic domain of Toc64 is digested^25^ while the membrane-inserted part remains intact. Toc75 remains mostly intact as it is deeply embedded within the bilayer, exposing only short loops to the cytosol^26,27^. N-terminal Edman sequencing of the immunoprecipitated larger fragment of OEP21 (Fig. 2b and Extended Data Fig. 2b) unambiguously identified a trypsin cleavage site in L5 (Fig. 2c)^5^, indicating that L5 is oriented toward the cytosol, and revealed that the N- and the C-termini are both located in the IMS. Trypsin-digestion experiments with recombinant OEP21 reconstituted in liposomes showed an identical cleavage pattern (Fig. 2a, right), suggesting that refolded OEP21 adopts a native topology. The addition of ATP or GAP to the liposome preparations slightly protected L5 from proteolytic digestion (Extended Data Fig. 2c), implying that the molecules interact with the loop. However, the orientation of OEP21 in liposomes could not be controlled, giving rise to only partial cleavage, whereas complete cleavage was observed after liposome disruption by the addition of detergent (Extended Data Fig. 2d).

**Extended Data Fig. 3:**
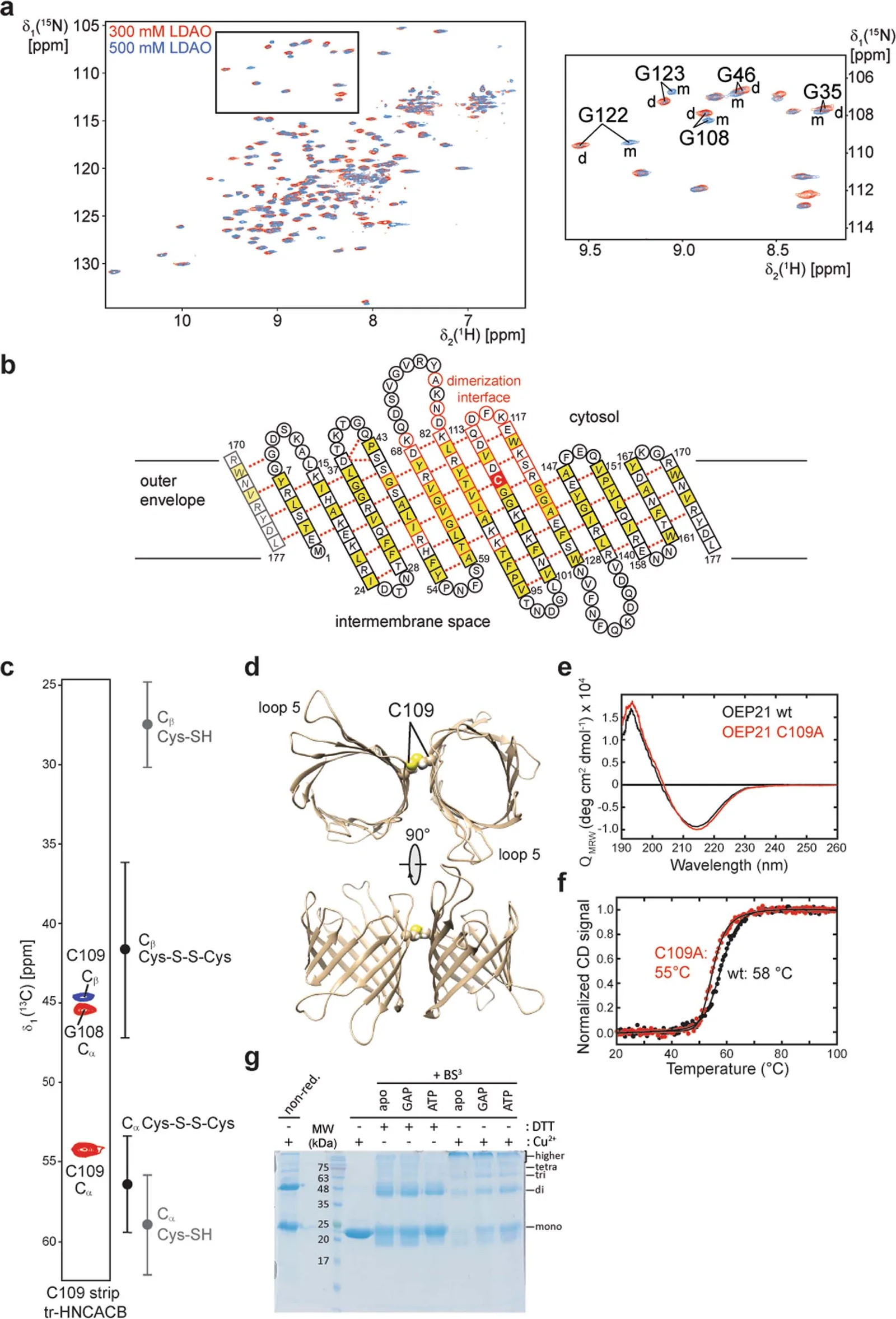
Detection and analysis of OEP21 dimerization in LDAO micelles. **(a)** Left: 2D-[^15^N,^1^H]-TROSY NMR experiments at the indicated concentrations of LDAO. Right: Glycine region of the TROSY spectrum (see box) with the monomeric (m) and dimeric (d) species labeled in each case. **(b)** Residues experiencing pronounced chemical shift perturbations are marked in red in the topology plot. The long β-strands on one side of the β-barrel located around cystein 109 (shown as a filled red box) are strongly affected, suggesting that Cys109 can engage in an inter-monomer disulfide bridge. Hydrophobic amino acids are indicated by yellow boxes. **(c)** Characteristic NMR chemical shifts for the C_α_ and C_β_ resonances^28^ of Cys109 as detected in 3D-tr-HNCA (C_α_ signals in red) and tr-HN(CA)CB (C_β_ signal in blue) experiments indicates the presence of a disulfide bridge in OEP21. **(d)** Structural model of disulfide-bridged OEP21 as obtained by manual docking, disulfide bond generation and a subsequent equilibration in a 100 ns MD simulation in a DMPC/DMPG lipid bilayer membrane. **(e)** A comparison of the far-UV CD spectra of OEP21 wt (black) and C109A (red) and **(f)** CD-detected thermal stability of C109A mutant (55 °C, red) as compared to wt-OEP21 (58 °C, black) at 215 nm. **(g)** Analysis of oligomerization of OEP21 in monomeric state (+ DTT) or preformed dimers (+ Cu^2+^) and the effect of GAP or ATP. ‘non-red.’ sample lane shows the Cu^2+^-oxidized protein prior to BS^3^ application which was ran under non-reducing conditions. Figure (g) is representative of n = 3 independent experiments. Source data

Crosslinking experiments have shown that OEP21 forms larger oligomers in the OE^9^. To corroborate this finding, we performed Blue Native (BN)-PAGE (Extended Data Fig. 2e,f) of OEVs extracted with the mild detergent DDM, in which OEP21 oligomers could be detected in a molecular weight range of 40 to approximately 200 kDa, confirming the presence of dimers and higher-order oligomers in a native membrane environment. Chemical crosslinking experiments with recombinant OEP21 in liposomes (Fig. 2d) or LDAO detergent micelles (Extended Data Fig. 2g) also showed the presence of higher-order oligomers. ATP, which stabilizes OEP21, led to slightly reduced oligomer formation. Furthermore, the oligomeric state of OEP21 can be controlled by adjusting the detergent concentration (Extended Data Fig. 2h). To estimate the impact of oligomerization on the OEP21 structure, we recorded 2D-[^15^N,^1^H]-TROSY NMR experiments at a 100- or 300-mM detergent concentration (Fig. 2e). At the lower concentration, we observed strong line broadening of the backbone amide resonances in the folded β-barrel, as expected for a larger assembly. However, strong NMR signals originating from L5 were visible in the oligomeric state, suggesting that the cytosolic loop is affected by oligomerization. In addition, signals in the random coil chemical shift region appeared in the spectrum, possibly caused by squeezing of the β-barrel in the oligomer. Interestingly, the addition of ATP improved the NMR spectral quality and resulted in the disappearance of the random coil peaks, confirming the assumption that ATP can stabilize the shape of the OEP21 β-barrel and reduce its oligomeric state. Using isothermal titration calorimetry (ITC) experiments, we showed that OEP21’s affinity for ATP decreases at detergent conditions that favor the oligomer, presumably owing to decreased accessibility of the binding site (Fig. 2f). A comparison of the 2D-[^15^N,^1^H]-TROSY spectra of OEP21 at 300 and 500 mM LDAO (Extended Data Fig. 3a,b) showed spectral changes at one side of the β-barrel, likely indicating the dimerization surface. Interestingly, a cysteine residue (Cys109) in the center of this region is well-positioned to engage in a disulfide bridge. An analysis of Cu^2+^-oxidized OEP21 by SDS–PAGE clearly showed that cysteine-mediated crosslinking is possible (Fig. 2g). In addition, NMR ^13^C_α_ and ^13^C_β_ chemical shifts of Cys109 in OEP21 indicate the presence of a disulfide bridge^28^ (Extended Data Fig. 3c), which enabled the assembly of a dimeric disulfide-bridged structural model of OEP21 (Extended Data Fig. 3d and Supplementary Data 1). Changing Cys109 to alanine does not alter the secondary structure content of OEP21 but leads to a reduction in its thermal stability (Extended Data Fig. 3e,f), demonstrating the importance of the cysteine residue. Strikingly, we observed a severe enhancement in oligomerization when Cys109 is oxidized, whereas GAP or, more prominently, ATP reduced this process (Extended Data Fig. 3g).

### OEP21 binds to metabolites by a promiscuous electrostatic mechanism

**Fig. 3:**
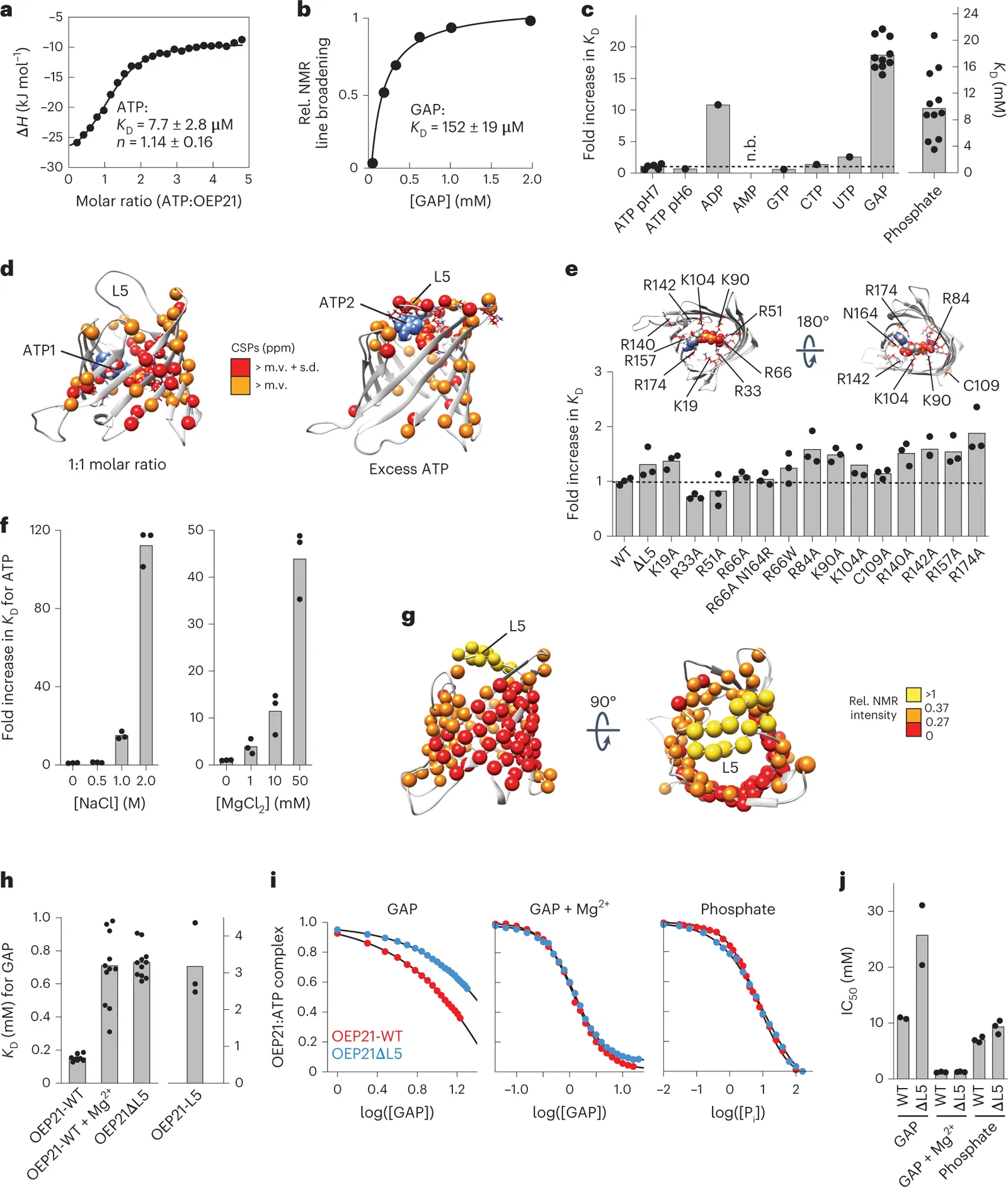
Metabolites bind to OEP21 in a charge-dependent and competitive manner. **a**,**b**, ITC (**a**) and NMR (**b**) binding experiments with OEP21 and ATP or GAP. *ΔH*, binding enthalphy. **c**, Affinities of OEP21 for negatively charged metabolites. Bars indicate mean value of individual measurements, which are multiple ITC or FP experiments (*n* ≥ 2) or individual residues from the NMR titration experiment (*n* = 12). n.b., no binding could be detected by ITC for AMP. **d**, NMR chemical shift perturbations mapped onto an MD-based complex structural model of OEP21 and ATP at an internal, high-affinity (ATP1) and a peripheral, low-affinity (ATP2) binding site. m.v., mean value; s.d., standard deviation. **e**, Relative *K*_D_ values of OEP21 variants without L5 or containing single point mutations of positively charged residues obtained from *n* = 3 fluorescence polarization (FP) measurements with MANT-ATP. **f**, Effect of NaCl and MgCl_2_ concentration on the affinity between OEP21 and ATP, measured by FP. Bars represent mean value of *n* = 3 individual measurements. **g**, Relative NMR signal intensities of OEP21 upon the addition of 5 mM GAP mapped onto the OEP21 structure. **h**, The affinity of OEP21 for GAP in the presence of Mg^2+^or with deletion of L5. OEP21 L5 alone weakly interacts with GAP, as probed by NMR. **i**, Competition experiments with a complex of MANT-ATP and OEP21-WT or OEP21ΔL5 upon stepwise addition of GAP, GAP + Mg^2+^, or phosphate. **j**, IC_50_ values derived from the experiments shown in **i**. Bars in **h** and **j** represent mean values of *n* ≥ 2 measurements. Source data

**Extended Data Fig. 4:**
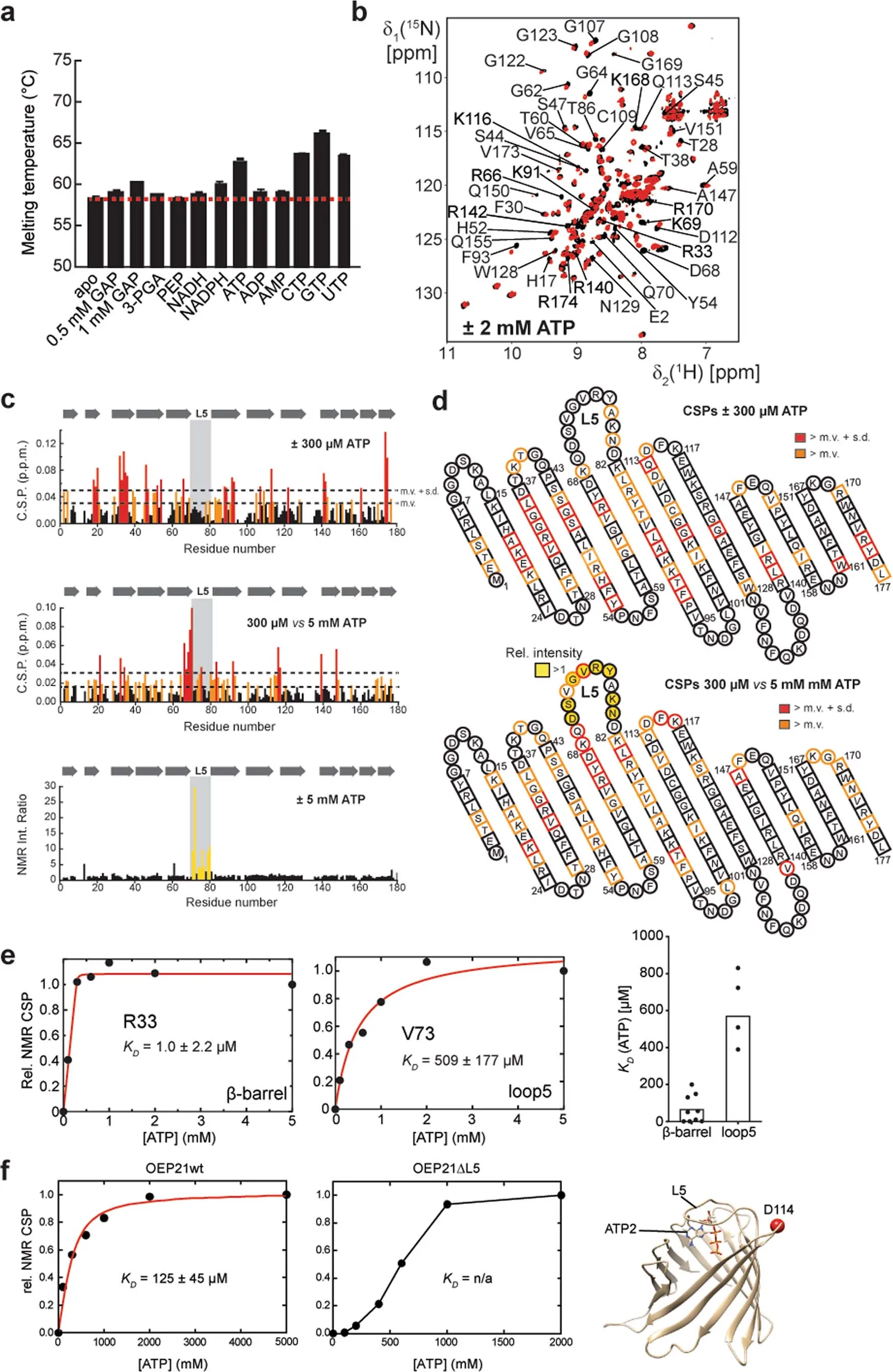
Stability of OEP21 in the presence of metabolites and NMR titrations with ATP. **(a)** CD-detected thermal stabilities of OEP21 measured at 215 nm in 10 mM sodium phosphate pH 7.0, 0.5 mM DTT and 0.1 % LDAO in presence of 0.5 mM metabolites. For GAP, 0.5 and 1 mM concentrations were used, as indicated. Error bars represent the error from curve fitting. **(b)** 2D-[^15^N,^1^H]-TROSY NMR spectra of 300 µM ^2^H,^15^N-labeled OEP21 in 20 mM HEPES pH 7.0, 50 mM NaCl, 0.5 mM EDTA, 5 mM DTT and 300 mM LDAO with and without the addition of 2 mM ATP, measured at 950 MHz proton frequency and 303 K. **(c)** CSPs within ^2^H,^15^N-labeled OEP21 upon the addition of 300 μM (top) and 5 mM ATP (bottom) and their comparison (middle). **(d)** Topology plots for CSPs shown at **(c)**. **(e)** Dissociation constants for ATP derived from NMR CSP titration data per individual amino acid residues of OEP21 located in the β-barrel pore or L5. Plots of the residues, R33 and V73, were shown as representative plots for β-barrel pore and L5, respectively. The bar diagram shows the average *K*_*D*_ values for ATP in the β-barrel and loop regions. **(f)** NMR titration of D114 with ATP in the presence (OEP21 wt) or absence (OEP21ΔL5) of the loop region. Source data

It has been suggested that ATP can inhibit TP binding to OEP21 (ref. ^9^). To reveal the molecular and structural basis for the interaction of OEP21 with metabolites, we determined the binding affinities of relevant negatively charged molecules by a combination of ITC and NMR to characterize high- and low-affinity interactions, respectively. ATP, carrying four negative charges at physiological pH, shows an affinity in the low µM range, with a 1:1 binding stoichiometry (Fig. 3a). By contrast, as probed by NMR, the monophosphorylated substrate GAP binds with a much lower affinity of ~150 µM (Fig. 3b). To identify the molecular features that are required for binding to OEP21, we determined the affinities of a larger pool of metabolites (Fig. 3c). The high affinity for ATP was not altered by changes in the pH, whereas the number of phosphate moieties in the metabolite appeared to be more critical: the affinity for ADP^3−^ was markedly reduced, and no binding could be detected for AMP^2−^ by ITC, suggesting an equally weak interaction as that observed for GAP (Fig. 3b). Other triphosphate nucleosides (GTP, UTP, CTP) showed a similar binding affinity to that of ATP, with a slightly lower affinity for the smaller pyrimidine nucleotides (UTP and CTP). These data suggest that binding of metabolites to OEP21 is dominated by their negative charge density. However, in addition to the phosphate moiety, other parts of the metabolite contribute to the interaction, as is evident from the large difference in affinity of GAP and phosphate (Fig. 3b,c). In line with the binding data, the stabilization of OEP21 was stronger with molecules carrying a higher number of negative charges (Extended Data Fig. 4a). To obtain higher-resolution insights on the ATP-binding mode, we next conducted NMR titration experiments and analyzed the ligand-induced chemical shift perturbations in OEP21 (Fig. 3d and Extended Data Fig. 4b). We identified a binding site located within the β-barrel pore that was fully occupied at a 1:1 protein-to-ATP molar ratio. At excess ATP, CSPs and an increase in NMR signal intensity were visible at the cytosolic entry of the pore, involving L5 (Fig. 3d and Extended Data Fig. 4c,d). The affinity of the binding site inside the pore was in the low µM range, in good agreement with the ITC data, whereas the peripheral binding site exhibits an average dissociation constant (*K*_D_) value of ~500 µM (Extended Data Fig. 4e), which could not be detected by ITC. Furthermore, deletion of L5 in OEP21 (ΔL5) led to a reduced affinity for ATP at the exterior binding site (Extended Data Fig. 4f).

**Extended Data Fig. 5:**
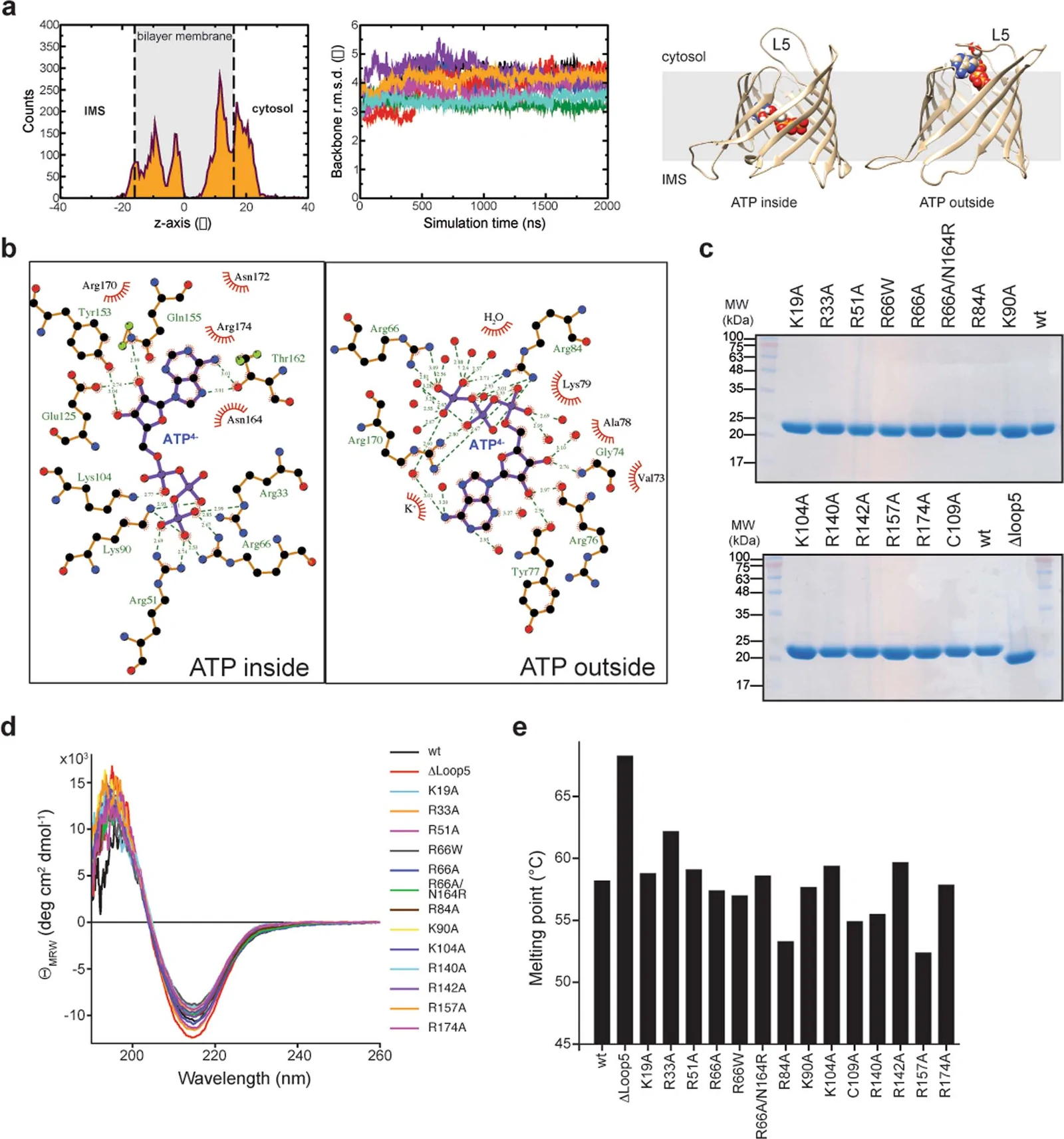
Interaction of ATP with OEP21 obtained from MD simulations and characterization of the produced OEP21 variants. **(a)** (left) Analysis of the population densities of ATP from MD simuations along the OEP21 channel. Negative values along the pore axis indicate the IMS and positive values the cytosol. The two broken vertical lines mark the lipid bilayer membrane region. (middle) Root mean square deviations (r.m.s.d.) within secondary structure elements of OEP21 in complex with ATP^4−^ (ATP 1–8, 2 µs each). (right) Representative binding poses of ATP to OEP21 inside the pore or at the peripheral binding site involving loop 5. **(b)** LigPlot^66^ representation of the interactions in each of the two binding poses. **(c)** Coomassie-stained SDS-PAGE of OEP21 variants show high protein purity. **(d)** CD spectra of 10 µM OEP21 variants in 10 mM Na-phosphate pH 7.0, 0.5 mM DTT and 0.1 % LDAO. The β-sheet content of the Δloop5 (red curve) variant is highest due to the missing loop. **(e)** CD-detected thermal melting points of the samples shown in **(c)** detected at 215 nm. Source data

To obtain additional details on the binding mode of ATP, we conducted unrestrained MD simulations of up to 2 µs in duration, until a stable binding pose was reached (Extended Data Fig. 5a, left, and Supplementary Methods). This unbiased MD approach showed that two basic binding poses can be adopted, one inside the β-barrel and the second at the peripheral binding site (Extended Data Fig. 5a, right). In the internal binding pose, ATP has a multitude of bonding interactions with OEP21, whereas fewer contacts are formed in the peripheral location, corroborating the observed differences in affinity (Extended Data Fig. 5b). The two binding poses are in excellent agreement with the experimental NMR CSP pattern of OEP21 with ATP (Fig. 3d). However, the MD data and the observed global NMR effects suggest that more than just a single binding pose might be possible for each site. To investigate the binding specificity and the impact of positive charges in OEP21, we produced 14 point variants in regions identified by the NMR titrations, as well as the ΔL5 variant (Extended Data Fig. 5c). All OEP21 variants were properly folded and showed comparable stability (Extended Data Fig. 5d,e). Next, we probed the binding affinity of these variants by fluorescence polarization (FP) experiments with MANT-ATP. As shown in Figure 3e, the *K*_D_ values for ATP determined with this set of proteins all lie within 50% of the value obtained with the wild-type (WT) protein. This implies that the removal or shifting (with the R66A N164R variant) of individual positive charges can be compensated by other positively charged side chains in close proximity. Furthermore, deletion of L5 does not affect the high-affinity binding site inside the pore. These data suggest a promiscuous binding mechanism that is largely dependent on the bulk electrostatic properties of the OEP21 pore. To further validate this conclusion, we performed ATP-binding assays at increasing NaCl concentrations (Fig. 3f, left) and observed an almost 120-fold drop in binding affinity at 2 M salt. Mg^2+^ is associated with ATP in the cell and leads to a reduction in its negative charge density. Consequently, binding assays in the presence of increasing MgCl_2_ concentrations indicated an almost 50-fold decrease in affinity at 50 mM MgCl_2_ (Fig. 3f, right).

**Extended Data Fig. 6:**
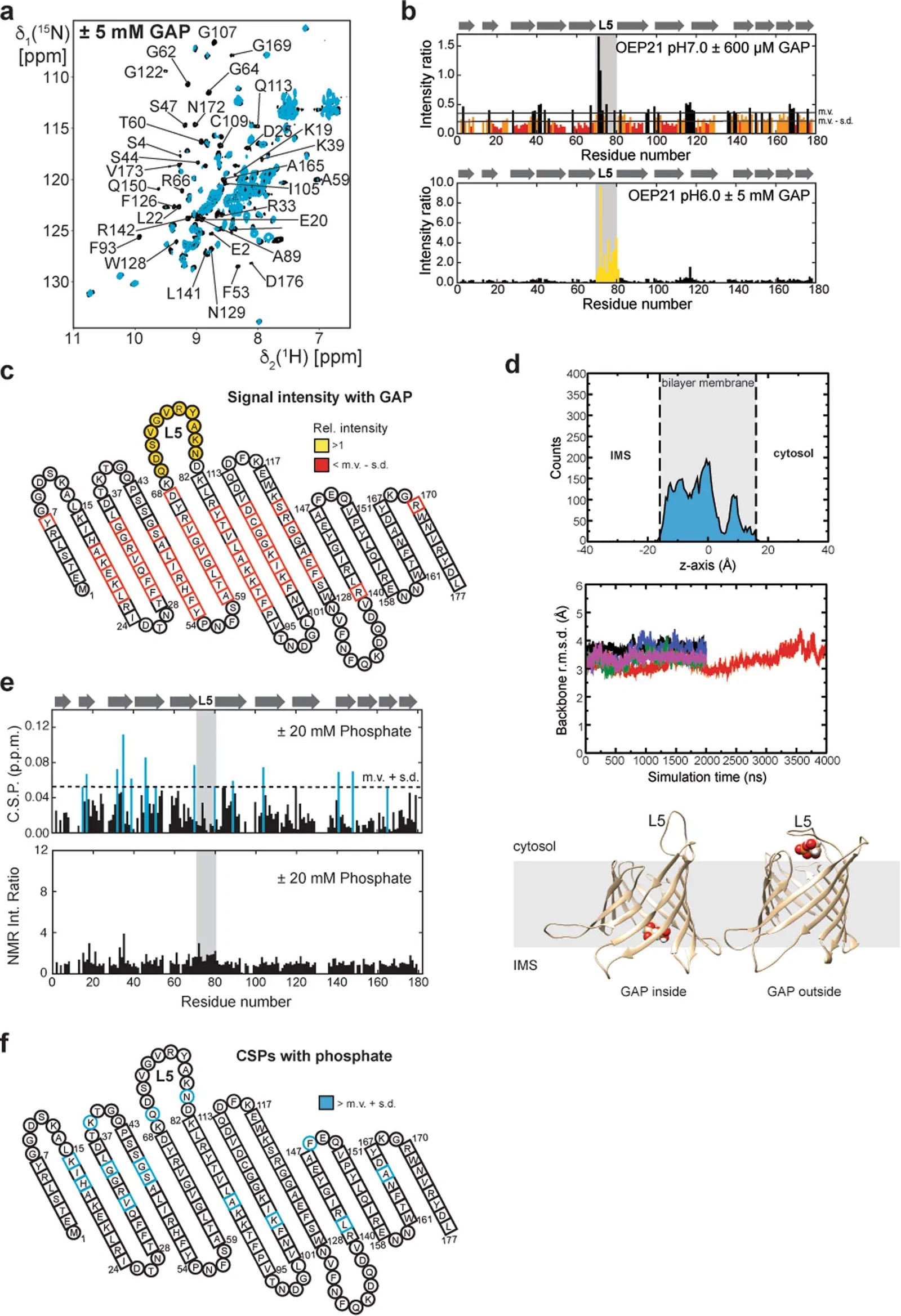
NMR titrations of OEP21 with GAP and phosphate and analysis of GAP interaction by MD simulations. **(a)** 2D-[^15^N,^1^H]-TROSY NMR spectra of 300 µM ^2^H,^15^N-labeled OEP21 in 20 mM HEPES pH 7.0, 50 mM NaCl, 0.5 mM EDTA, 5 mM DTT and 300 mM LDAO with and without the addition of 5 mM GAP, measured at 950 MHz proton frequency and 303 K. **(b)** Intensity ratio of signals in the 2D-[^15^N,^1^H]-TROSY NMR spectrum of ^2^H,^15^N-labeled OEP21 in the apo form or in presence of 600 µM or 5 mM GAP. **(c)** Data shown in **(b)** mapped on the topology plot of OEP21. **(d)** (top) The rather broad population densities of GAP inside the OEP21 pore indicate that GAP is loosely bound to the channel interior and periphery. Negative values along the pore axis indicate the IMS, positve values the cytosolic side. The two broken vertical lines mark the lipid bilayer membrane region. (middle) r.m.s.d within secondary structure elements of OEP21 in complex with GAP^2−^ (GAP1-5, 2 or 4 µs) show that its structure stays intact during the simulation. (bottom) Representative binding poses of GAP to OEP21 inside the pore or at the peripheral binding site involving loop 5. **(e)** Chemical shift perturbations and signal intensity ratios of the NMR signals of OEP21 upon the addition of 20 mM phosphate extracted from 2D-[^15^N,^1^H]-TROSY NMR experiments. **(f)** Blue bars in **(e)** color-coded on the topology plot of OEP21.

**Extended Data Fig. 7:**
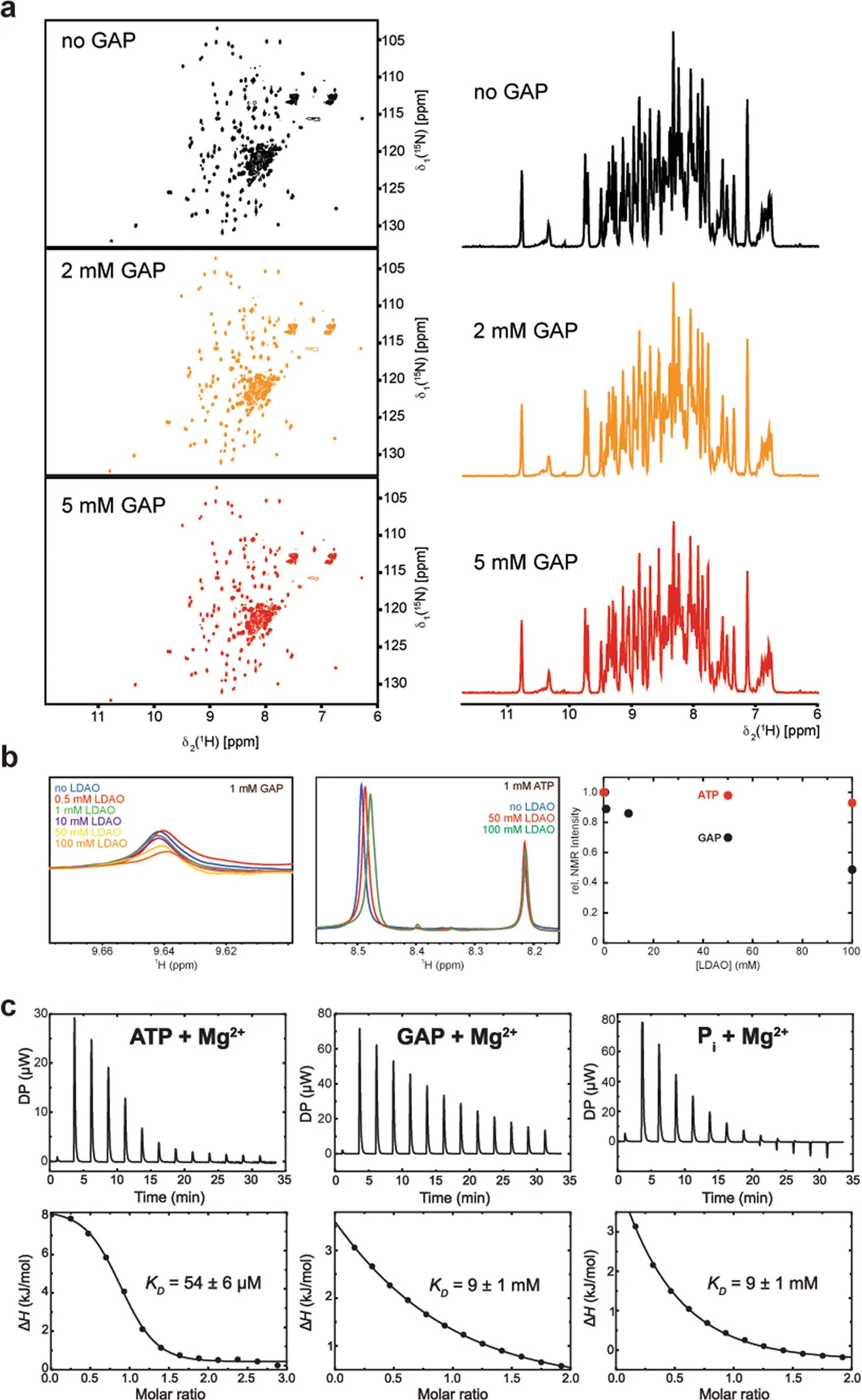
Control measurements for the interaction of GAP and ATP with LDAO micelles and their affinities for Mg^2+^. **(a)** (left) 2D-[^15^N,^1^H]-TROSY NMR spectra of 400 µM ^2^H,^15^N-labeled OmpX in LDAO micelles in 20 mM sodium phosphate, pH6.0, 50 mM NaCl, 0.5 mM EDTA and the indicated concentrations of D-GAP at 30 °C and 1.2 GHz magnetic field. (right) 2D-projections along the ^1^H dimension of the 2D-spectra shown on (left). **(b)** (left) ^1^H NMR spectra of the proton of the aldehyde moiety of GAP (9.64 ppm ^1^H) at increasing LDAO concentration, as labeled. The decrease in signal indensity indicates an unspecific interaction. (middle) same as in (left) but with the protons in the nucleobase of ATP (8.48 ppm ^1^H) upon the addition of LDAO at a 50 and 100 mM concentration. No marked signal decrease is observed for ATP, suggesting that it does not interact with the micelle. (right) Relative NMR peak intensities derived from the data in (left) and (middle). Buffer: 10 mM Na-phosphate pH7.0, 20 mM NaCl, 7% (v/v) D_2_O. **(c)** (left) 10 mM MgCl_2_ was titrated into 1 mM ATP. (middle) 100 mM MgCl_2_ was titrated into 10 mM GAP. (right) 150 mM MgCl_2_ was titrated into 15 mM potassium phosphate (P_i_). Buffer conditions for all experiments were: 10 mM HEPES pH7.0, 20 mM NaCl. The binding stoichiometry (metabolite:Mg^2+^) is 1:1 for ATP and 1:2 for GAP and phosphate. Source data

Next, we used NMR to characterize the interaction between OEP21 and GAP, the primary product of photosynthesis. Owing to the weaker affinity than for ATP, the addition of GAP induced line broadening of the NMR signals in the β-barrel structure (Extended Data Figs. 6a–c) in a concentration-dependent manner. The amino acids that experience the most pronounced line broadening effects are located at the side of the β-barrel that is involved in dimerization (Extended Data Fig. 3a,b) and which has the highest density of positively charged residues (red spheres in Fig. 3g). To exclude nonspecific binding effects of GAP with the LDAO micelle as a reason for the observed line broadening, we recorded 2D-NMR spectra with the unrelated bacterial β-barrel membrane protein OmpX and did not observe any effect, even at a GAP concentration of 5 mM (Extended Data Fig. 7a). Next, we performed unrestrained MD simulations with GAP to explore its possible binding poses with OEP21 (Supplementary Methods). As expected, GAP binds to positively charged patches inside the pore but also interacts with cytosolic L5 (Extended Data Fig. 6d, bottom), a finding that is corroborated by an increase in the NMR signal intensity of residues in L5 upon the addition of GAP (Extended Data Fig. 6b, bottom). To quantify this finding, we next used NMR to derive affinities of GAP with the OEP21 β-barrel, L5 and OEP21ΔL5 (Fig. 3h). These data show that L5 is involved in GAP binding and causes approximately a fourfold increase in binding affinity. Furthermore, the addition of Mg^2+^ decreased the binding affinity to a similar extent. Residues in L5 interacted with GAP with lower affinity (~3 mM). As suggested by the lower binding affinity (Fig. 3c), the addition of phosphate did only cause NMR chemical shift perturbations but did not alter the intensity of residues in L5 (Extended Data Fig. 6e,f).

Next, we aimed to explore whether metabolites can bind to OEP21 in a competitive manner. We performed FP experiments in which GAP, GAP and Mg^2+^, or phosphate was added to a preformed complex between OEP21 (WT or ΔL5) and MANT-ATP (Fig. 3i). The addition of GAP led to a stepwise dissociation of MANT-ATP with a half-maximal inhibitory concentration (IC_50_) value of ~10 mM for OEP21-WT and ~26 mM for OEP21ΔL5 (Fig. 3j). Because Mg^2+^ is released from the chloroplast thylakoid lumen into the stroma under light conditions^29^, and Mg^2+^ transporters have been reported in the chloroplast IE^30^, it is very likely that elevated concentrations of both GAP and Mg^2+^ in the IMS are present. The simultaneous addition of GAP and Mg^2+^ to the OEP21–MANT-ATP complex decreased the IC_50_ values for both OEP21 constructs to ~1 mM. This behavior, together with the weaker binding affinity of ATP in presence of Mg^2+^, indicates that GAP and Mg^2+^ synergistically facilitate ATP dissociation from OEP21. Inorganic phosphate also dissociated the OEP21–ATP complex with an IC_50_ value of ~10 mM, again with a lower value for OEP21-WT than for the ΔL5 variant (Fig. 3i,j). Such a high phosphate concentration lies within what has been observed in plant cells (1 to 10 mM)^31^. In comparison, the relative high GAP concentrations required for ATP dissociation can be rationalized by partial unspecific binding to the detergent micelle, as probed by one-dimensional (1D) NMR (Extended Data Fig. 7b). As evident from our ITC experiments and literature reports^32^, Mg^2+^ has a strong affinity for ATP (50 µM) but interacts only weakly with GAP or phosphate (*K*_D_, ~9 mM each) (Extended Data Fig. 7c). These data suggest that phosphorylated metabolites utilize the same positively charged binding surface of OEP21 and that dissociation of the high-affinity binder ATP can be achieved at cellular solute concentrations.

### Size-selective metabolite translocation through OEP21

**Fig. 4:**
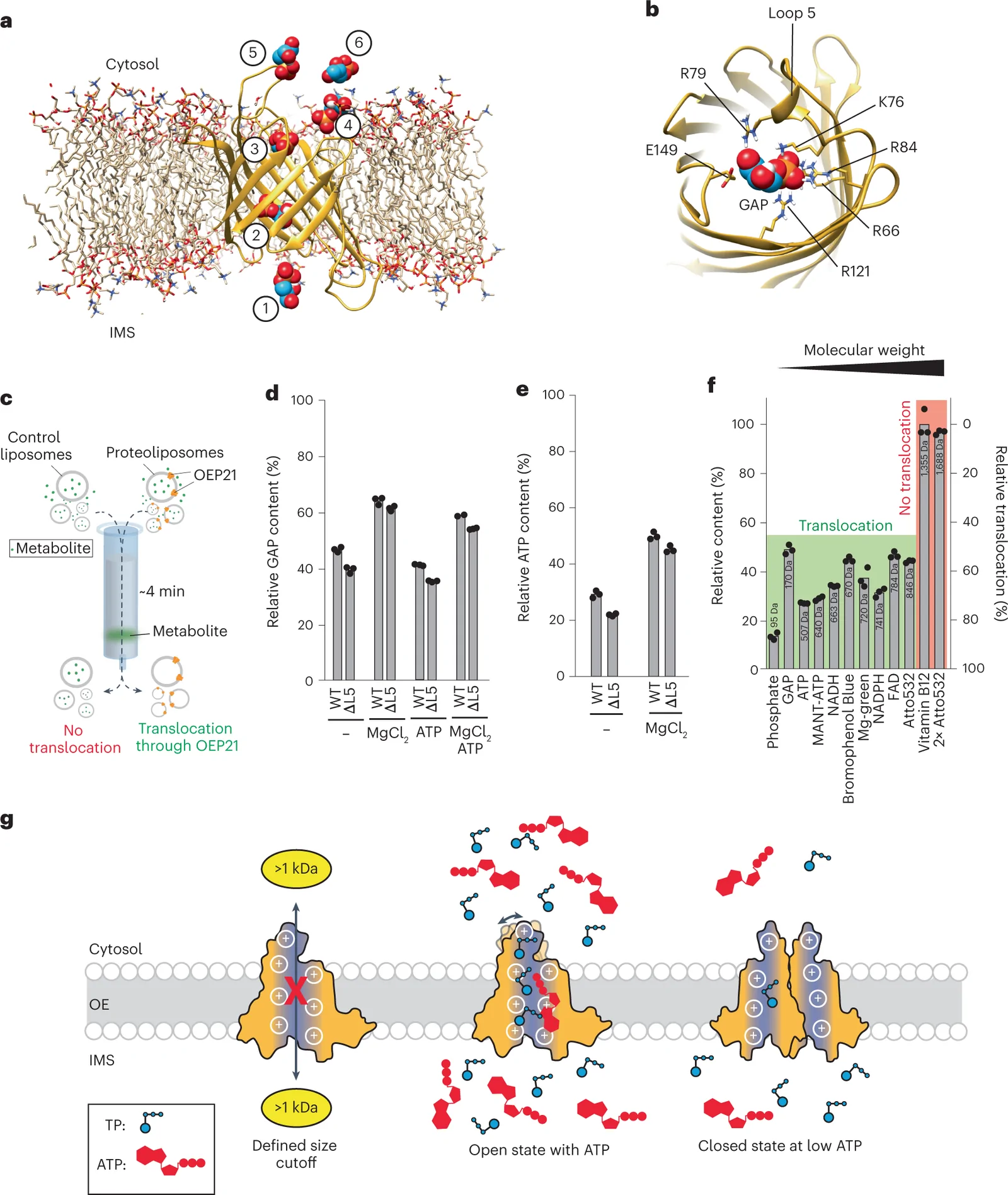
OEP21 is a dynamic pore that is stabilized by ATP and allows passage of small metabolites. **a**, Translocation trajectory of GAP through the OEP21 pore, obtained by MD simulations (numbers indicate the order of the binding poses in the trajectory). GAP transiently interacts with positive surface patches on OEP21 and finally binds to loop 5 before its dissociation. **b**, The external binding site is formed by positively charged residues of the pore and loop 5. **c**, Setup of the SEC translocation assay. **d**, GAP translocation is increased by ATP and is less efficient with MgCl_2_. Deletion of L5 slightly increased the translocation efficiency. **e**, The same as in **d**, but ATP translocation was monitored. **f**, Translocation assay with molecules of increasing molecular weight shows that the size cutoff is at ~1 kDa. Bars in **d**, **e** and **f** represent the mean value of *n* = 3 measurements. Each translocation assay was repeated *n* ≥ 2 times, with similar results. **g**, Schematic representation of OEP21 functionality. OEP21 has a substrate cutoff of 1 kDa. At physiological ATP concentrations, OEP21 is in its open state, whereas under oxidative stress and low ATP conditions, enhanced OEP21 oligomerization leads to pore closure. Source data

**Extended Data Fig. 8:**
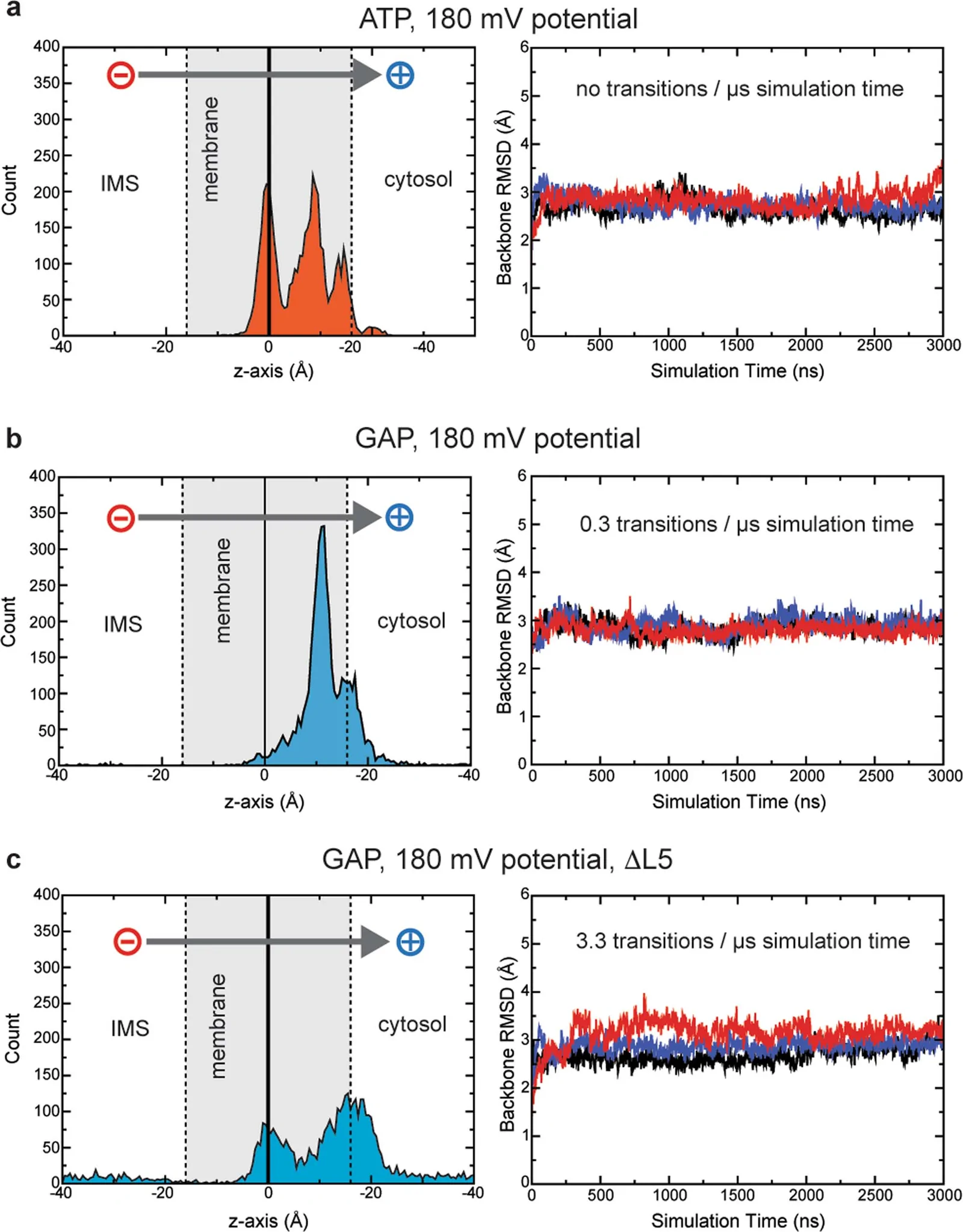
MD simulations with a membrane potential to observe GAP translocation across OEP21. **(a)** Analysis of the population densities of ATP during a 3 µs MD simulation shows that it interacts with the pore interior and L5. The simulation was stable as indicated by the constant r.m.s.d.values. No transition of ATP was oberved in this time scale. **(b)** same as in **(a)** but with GAP. Here, GAP translocation was observed. **(c)** same as in **(b)** but with OEP21ΔL5. Without L5 ~10-times more GAP translocation events are observed.

**Extended Data Fig. 9:**
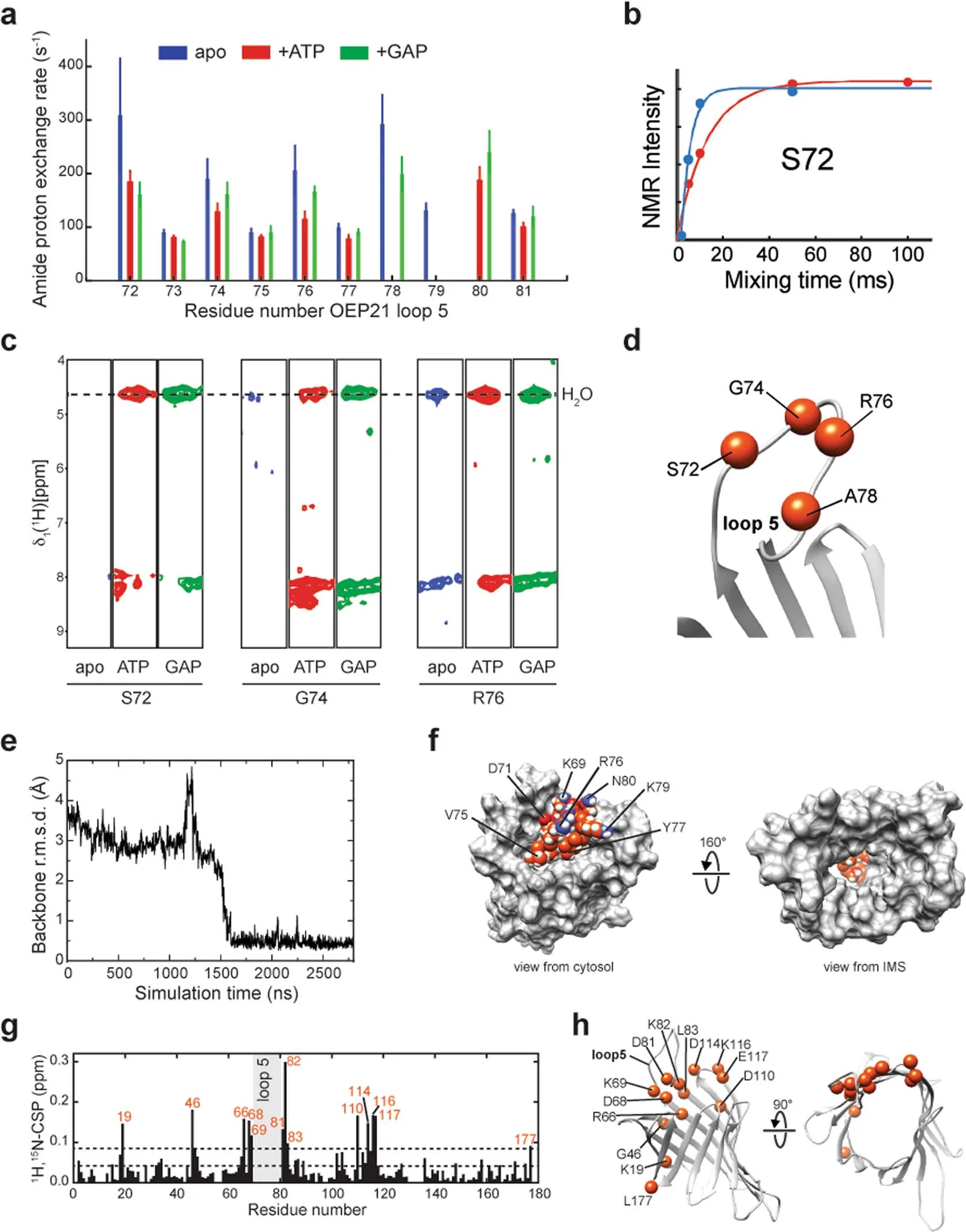
L5 participates in the OEP21 metabolite interaction and can transiently cover the OEP21 pore. **(a)** Amide proton exchange rates at 303 K and pH 6.0 for OEP21 L5 determined by CLEANEX NMR experiments^59^ are reduced if bound to 2 mM ATP or 5 mM GAP. Under these pH conditions, most of the NMR signals in a 2D-[^15^N,^1^H]-TROSY experiment can be observed for L5 even in the apo form, enabling a comparison. Error bars represent the errors obtained from curve fitting. Missing values are due to signal overlap or low peak intensity in the corresponding NMR spectra. **(b)** Representative builtup curve of the exchange experiment for residue S72 in the apo (blue) and ATP-bound (red) states. **(c)** Strips from a 3D-^15^N-edited-[^1^H,^1^H]- NOESY-TROSY experiment (40 ms mixing time) with ^2^H,^15^N-labeled OEP21 at 303 K and pH 6.0. Diagonal and crosspeaks are absent or very weak in the apo form but visible if bound to ATP or GAP. **(d)** Residues in L5 that show high exchange rates in the apo state and a marked reduction in the ligand-bound states. **(e)** r.m.s.d. of L5 backbone coordinates in OEP21 relative to the final pore-inserted state. Insertion of L5 into the pore occurs after ~1.5 µs and stays stably bound until the end of the simulation at 2.8 µs. **(f)** MD-derived structural model of the final pore-inserted state of L5 in OEP21. **(g)** NMR CSPs of OEP21 wt *versus* OEP21ΔL5 in the apo form. Significant CSPs (larger than the CSP mean value plus 1 standard deviation) are labeled. **(h)** Residues experiencing a significant CSP upon removal of L5 in the apo state are mapped onto the structure of OEP21. Not only residues adjacent to loop 5 are affected but also more remote regions (residues 19, 46 and 110-117), suggesting that loop 5 can sample multiple conformations and affect most parts of the cytosolic opening of the pore.

To gain further insights into the metabolite translocation pathway across OEP21, we conducted MD simulations, where GAP was initially placed at the entrance of the pore in the IMS. To facilitate translocation within a 3-µs simulation time, we applied a 180 mV membrane potential with a positive pole at the cytosolic side. The obtained trajectory shows that GAP is hopping along patches of positive charges on one side of the barrel and eventually sticks to the exterior binding site involving L5 (Fig. 4a and Supplementary Video 1) that provides two additional positively charged residues, Lys76 and Arg79 (Fig. 4b). Finally, GAP is released by an outward movement of L5. The interaction with L5 slows down translocation, which is supported by a simulated translocation frequency of GAP that is about ten times higher with OEP21ΔL5 (Extended Data Fig. 8). In line with the MD data, the amide hydrogen exchange rates in L5 are reduced in the complex with GAP and, more prominently, with ATP (Extended Data Fig. 9a–d), confirming that L5 interacts with negatively charged metabolites. MD simulations of OEP21 in the apo state show that L5 can transiently cover the pore in a µs time scale, which was confirmed by the observation of NMR chemical shift perturbations between OEP21-WT and OEP21ΔL5 only in close proximity to L5 (Extended Data Fig. 9e–h).

**Extended Data Fig. 10:**
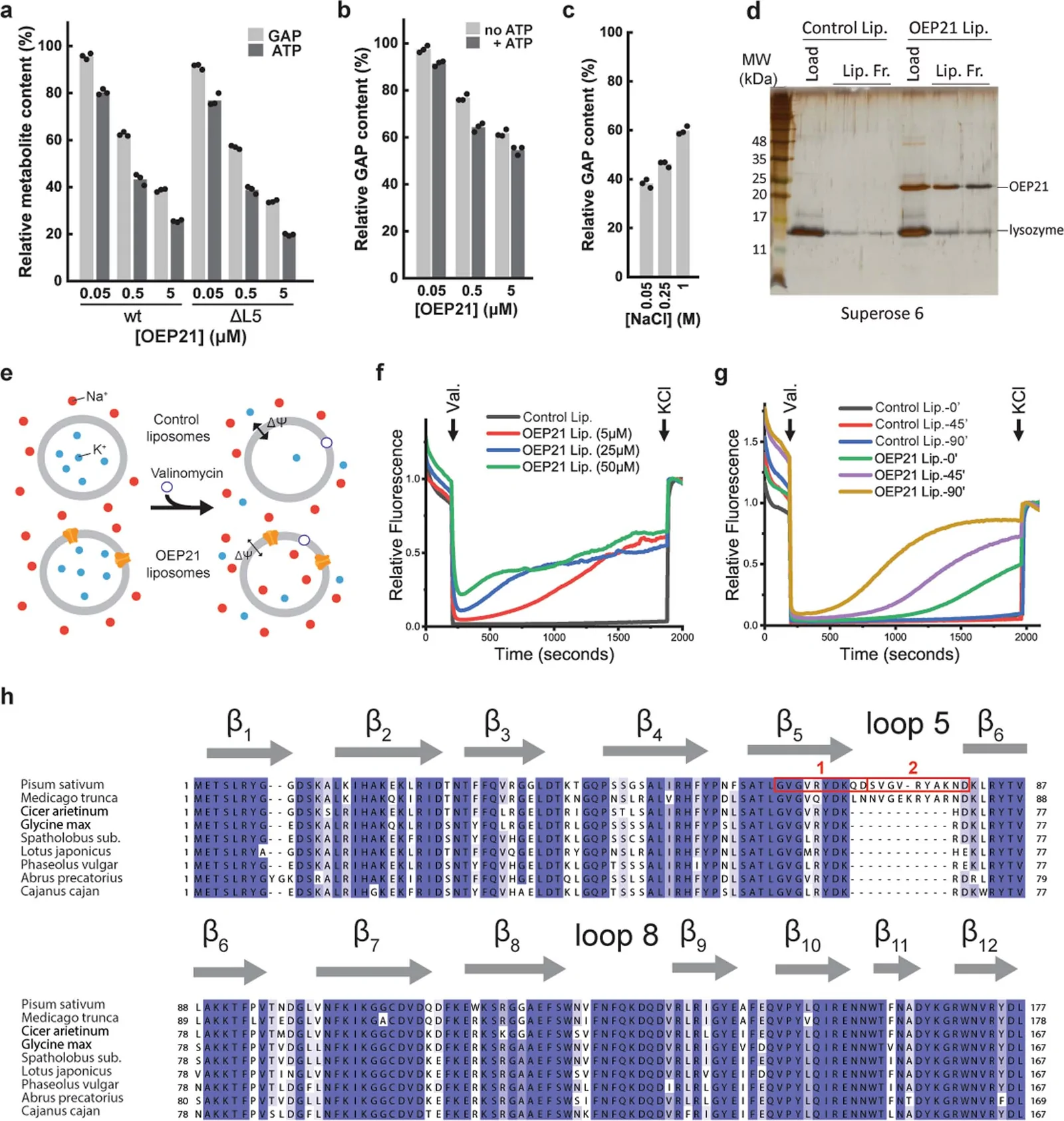
Analysis of the functionality of OEP21 using SEC and membrane potential measuments, and multiple sequence alignment of OEP21 from different species. **(a)** Relative amount of GAP or ATP in OEP21 proteoliposomes subjected to PD10 size exclusion chromatography at increasing OEP21 wt (left) and ΔL5 (right) concentrations. **(b)** GAP translocation assay with liposomes prepared in absence (light gray) and the presence (dark gray) of 2 mM ATP. **(c)** GAP translocation assay in the presence of increasing NaCl concentrations. The indicated data points at (a), (b) and (c) show three technical replicates of a representative experiment and bars show mean of the replicates. Similar results were obtained at least n ≥ 2 independent experiments. **(d)** SDS-PAGE of lysozyme loaded control (left) and OEP21 containing (right) liposomes applied to Superose 6 SEC column. Similar results were obtained for n = 2 independent experiments. **(e)** Schematic representation of the ΔΨ experiments. K^+^ ion specific ionophore valinomycin allows its passage from the liposomes resulting in generation of ΔΨ due to ionic imbalance across the liposomal membrane. Control liposomes keep ΔΨ by not allowing Na^+^ leakage into liposomes while OEP21 liposomes lose it due to passage of Na^+^ ions through the pore of OEP21. **(f)** Increasing OEP21 concentration cause faster loss of ΔΨ. Liposomes prepared in the presence of 250 mM KCl were diluted (1:1000) in the buffer containing 250 mM NaCl and the loss of potential measured directly after dilution utilizing fluoresence of potentiometric dye DiSC_3_(5). **(g)** Control liposomes or liposomes containing 5 µM OEP21 were diluted in the NaCl buffer as in **(f)** and they were masured either directly after dilution (0′) or after indicated time points (45′ and 90′). **(h)** Multiple sequence alignment of OEP21 from various C_3_ plants. In this compilation, L5 is only present in pea (*Pisum sativum*) and clover (*Medicago trunca*) (first two sequences from the top). All other parts of OEP21 show very high sequence conservation. A sequence analyis also suggests that loop 5 is a duplication of a directly adjacent peptide strech (aa 61–71), as indicated by the red boxes and numbers. Source data

Finally, we experimentally investigated the channel functionality by metabolite translocation assays using OEP21 proteoliposomes. For this purpose, metabolite-filled liposomes were subjected to size-exclusion chromatography (SEC), and the loss in metabolite content was quantified by comparing liposomes with and without increasing amounts of OEP21 (Fig. 4c). In the deadtime of the assay, which was ~4 min, GAP and ATP both passed through the channel (Extended Data Fig. 10a), even if both molecules were added at the same time. GAP translocation assays in the presence of 5 mM ATP inside and outside the liposomes show that GAP transport is slightly enhanced by ATP (Fig. 4d and Extended Data Fig. 10b), suggesting an activating, rather than an inhibitory, role for ATP. As proposed by MD simulations, deletion of L5 slightly increased the translocation activity of OEP21, leading to a lower metabolite content than with the WT protein (Fig. 4d). The addition of MgCl_2_ or elevated concentrations of NaCl to the transport assay (Fig. 4d and Extended Data Fig. 10c) resulted in reduced GAP translocation, in accordance with a reduced binding affinity (Fig. 3f,h). A similar behavior was observed for the translocation of ATP, where deletion of L5 increased and the addition of Mg^2+^ decreased the translocation efficiency (Fig. 4e). Despite their competitive binding modes to OEP21, GAP and ATP appeared to permeate the channel simultaneously. Thus, we next explored the substrate selectivity of OEP21 and the size limit of small molecules for channel permeation using translocation assays with molecules of increasing molecular weights (Fig. 4f). We demonstrate that molecules up to a molecular weight of ~1 kDa can pass the channel to a similar degree. However, larger molecules, such as vitamin B_12_, the dimeric fluorescent dye ‘2× Atto532′ (Fig. 4f) or the ~14-kDa protein lysozyme (Extended Data Fig. 10d) cannot pass. All investigated permeating molecules have a negative charge, except for bromophenol blue, for which a lower translocation efficiency was observed. These results clearly show that the specificity of metabolite translocation across OEP21 is broad, but with a sharp size limit, and there is a preference for negative charge.

Thus, we checked the degree of leakiness of OEP21 proteoliposomes for positively charged Na^+^ ions using valinomycin-dependent membrane potential (Δ*Ψ*) measurements with the fluorescent dye DiSC_3_(5) (Extended Data Fig. 10e). A membrane potential of 177 mV was stable in control liposomes, whereas, in OEP21 proteoliposomes, the potential was quenched in a time- and OEP21-concentration-dependent manner (Extended Data Fig. 10f), owing to Na^+^ leakage. Na^+^ leakage through OEP21 also occurred in the absence of a Δ*Ψ* (Extended Data Fig. 10g), but the kinetics were very slow. Thus, the translocation process of small cations is markedly slower than of much larger but negatively charged molecules, where almost complete release is already achieved after ~4 min (Fig. 4g).

## Discussion

In this study, we present the high-resolution NMR structure of the chloroplast OE membrane channel OEP21, which adopts a β-barrel topology with an overall cone-like shape (Fig. 1e). The highly positively charged pore interior surface (Fig. 1f) suggests a preferred translocation of negatively charged molecules, such as phosphate, TPs or ATP, as suggested by electrophysiology^9,14^. The OEP21 funnel in the OE is oriented with its wide opening toward the IMS (Fig. 2a–c), confirming its previously proposed outward-rectifying properties^9^. However, the translocation process and its directionality is most likely driven passively by a metabolite concentration gradient across the OE^6,9,14^. Furthermore, metabolite binding to OEP21 is promiscuous and dominated by the bulk electrostatic properties of the channel (Fig. 3), where the positively charged interior surface mediates binding of metabolites in a competitive manner (Fig. 3i,j).

OEP21 has a strong tendency to form oligomers (Fig. 2d and Extended Data Fig. 2), which induces conformational changes that lead to pore closure (Fig. 2e,f). However, the availability of ATP stabilizes the pore in a more open state (Fig. 4d). This tendency becomes even more pronounced at oxidizing conditions, when disulfide-bridged dimers are present (Extended Data Fig. 3), suggesting that oxidative stress conditions in plants might promote OEP21 closure. An important oxidative stress pathway is the hypersensitive response (HR), a type of programmed cell death to resist pathogen infections in plants^33^. Intriguingly, ATP levels have been shown to play a role in disease resistance and decrease dramatically during the HR^34^, which both would affect the structural state of OEP21.

Using NMR and MD simulations, we show that translocation of GAP across OEP21 occurs in the µs-to-ms time scale and takes place through transient interactions with positively charged patches inside the pore (Fig. 4a). L5 of OEP21 participates in the formation of an external binding site for negatively charged metabolites before their release into the cytosol (Fig. 4b). However, the presence of this loop does not seem to be essential for OEP21 functionality, because it is absent in most plants, with an overall high sequence conservation in other parts of the protein (Extended Data Fig. 10h). This finding suggests that L5 might be involved in the fine-tuning of metabolite flux in specific plants, such as pea and clover. Their genome is large and evolving faster than those of other members of Leguminosae^35^. Indeed, a sequence analysis suggests that L5 was generated by a duplication of a peptide stretch in the N-terminally-adjacent β-strand (Extended Data Fig. 10h). In line with this observation, deletion of L5 in pea OEP21 leads to more efficient GAP translocation, as observed in MD simulations and liposome assays (Fig. 4c–e and Extended Data Fig. 10a–c).

The large set of data allowed us to propose a functional model of OEP21 (Fig. 4g). OEP21 is dynamic in its apo state, allowing for conformational changes, such as squeezing of the β-barrel in the oligomeric state that is largely present in the lipid membrane. Such a shape change might lead to OEP21 closure, as supported by our binding and transport experiments, and as suggested for the mitochondrial porin VDAC^36^. ATP leads to a stabilization of the pore shape, keeping the channel in an active and permeable state. The µM affinity of ATP does not prevent binding and translocation of GAP. Whether ATP can slow down the kinetics of GAP translocation cannot be probed with our assay at the given time resolution, but the performed endpoint analysis after 4 min indicates that simultaneous translocation is possible. These data suggest a regulatory role of ATP for OEP21 functionality to enable TP transport under conditions where triose phosphate utilization (TPU), such as sucrose synthesis, is not limiting—a condition with a sufficiently high phosphate concentration inside the chloroplasts to enable ATP production and CO_2_ fixation. However, if TPU is limiting, i.e. the phosphate level is dependent on its regeneration, the ATP levels drop significantly, leading to OEP21 closure and restriction of further TP export to prevent depletion of phosphate inside chloroplasts, which would be detrimental for the plant cell^37^.

Besides the preference for negatively charged molecules, the main specificity of OEP21 appears to be that larger molecules cannot permeate. This finding suggests that the flux of larger molecules is most likely directed through the larger OEP family members, such as OEP24, OEP37 and OEP40. Proteomics data of plants with different levels of photosynthetic activity, for example, C_3_ versus C_4_ plants, show that the abundance of individual OEP family members is biased toward the larger pores if photosynthesis is more efficient^13^. Thus, not only the size limit, but also the overall transport capacity for metabolites, might be enhanced for larger OEPs. Compared with the outer mitochondrial membrane, where only three VDAC isoforms^38^ mediate metabolite exchange^39^, the situation seems to be more variable and fine-tuned in chloroplasts, where larger alterations in metabolite flux occur in different plants, requiring a variable set of OEPs. A potential functional overlap between specific OEP family members might be the main reason for the lack of a phenotype under normal growth conditions^12^. Thus, further investigations on the structural features and substrate specificities of other OEPs will be essential to uncover the functional connections within this poorly investigated protein family and evaluate the level of regulation and specificity of the network of channels in the chloroplast outer envelope.

## Methods

### Protein production and purification

The gene encoding pea OEP21 (GeneBank: AJ009987.1) was inserted into a modified pET21a vector (Amp^R^, Merck) by PCR-based restriction-free cloning, resulting in OEP21 harboring a non-cleavable C-terminal 10×His tag. For protein production, *Escherichia coli* BL21(DE3) were transformed with the described plasmid and grown to an optical density at 600 nm (OD_600_) of 0.6–0.8 at 37 °C. At this point, protein production was induced by the addition of 1 mM IPTG, and cells were shaken for another 4–5 h at 37 °C, collected by centrifugation (6,000*g*, 15 min, 4 °C) and stored at −80 °C. For lysis, cells were resuspended and homogenized in buffer A (50 mM Tris pH 8.0, 100 mM NaCl, 1 mM EDTA, 10 mM BME) + 1% Triton X-100, 0.2 mg ml^–1^ lysozyme (Sigma Aldrich) and one protease inhibitor tablet (Roche, cOmplete). Cells were sonicated (10 min, 30% amplitude, 1-s pulse, 2-s pause) and DNA was digested simultaneously by the addition of 1 U ml^–1^ DNase I (Roche) and 5 mM MgCl_2_. After centrifugation (38,769*g*, 20 min, 4 °C), inclusion bodies were washed with buffer A + 1% Triton X-100 and were centrifuged again. Inclusion bodies were finally dissolved in buffer B (6 M guanidinium chloride, 50 mM Tris pH 8.0, 100 mM NaCl, 10 mM imidazole and 5 mM BME), homogenized, centrifuged and filtered (0.45 μm) before further purification by Ni-NTA affinity chromatography. The supernatant was incubated for 1 h with the Ni-NTA resin (GE Healthcare), washed with 10 column volumes of buffer B and eluted with 10 column volumes of buffer B with 500 mM imidazole. The elution fraction was dialyzed overnight against 20 mM Tris pH 8.0, 50 mM NaCl, 1 mM EDTA and 2.5 mM BME in a 3.5-kDa molecular weight cutoff (MWCO) dialysis tube. Precipitated OEP21-10×His was dissolved in 6 M guanidinium chloride, 50 mM Na-phosphate pH 6.0, 100 mM NaCl, 5 mM EDTA and 10 mM DTT at a concentration of 5 mg ml^–1^. Protein refolding was performed by rapid dropwise dilution into a tenfold excess volume of refolding buffer consisting of 20 mM Na-phosphate pH 6.0, 50 mM NaCl, 1 mM EDTA, 3 mM DTT, 10% (v/v) glycerol and 0.5 % (m/v) LDAO, with moderate stirring at 4 °C for 2–4 h. To remove residual guanidinium chloride, dialysis (3.5-kDa MWCO) was done overnight in 20 mM Na-phosphate pH 6.0, 50 mM NaCl, 1 mM EDTA, 2.5 mM BME. Final purification was done by SEC (ÄKTA Pure system) using a Superdex 200 10/300 column (GE Healthcare) in 20 mM HEPES-KOH pH 7.5, 50 mM KCl, 0.5 mM EDTA, 5 mM DTT and 0.1% LDAO; 5 mM DTT was added to the sample before it was applied to the column. Protein samples were flash frozen and stored at −80 °C until further use. For buffer exchange, dialysis of the protein samples (6–8 kDa MWCO) in the corresponding buffer was performed.

Isotope-labeled protein (^2^H,^15^N; ^2^H,^15^N,^13^C; or selectively ILVAFY-labeled) was produced using published protocols^20^. In brief, for stereospecific Leu, Val methyl labeling, 300 mg L^–1^ stereospecific LV precursor ethyl 2-hydroxy 2-^13^C-methyl 3-oxobutanoate (according to established protocols^41,42^) and 80 mg L^–1^ of the Ile precursor α-ketobutyrate^43^ together with 2.0 g L^–1^ *d*_*4*_-succinate (Eurisotop or Sigma Aldrich) and 0.5 g L^–1^ 3-[^13^CH_3_]-2-*d*-l-Alanine (Eurisotop), as well as 80 mg L^–1^ of both uniformly ^15^N labeled l-Phe and l-Tyr (Sigma Aldrich), were added to the bacterial culture 1 h before induction of protein production with 1 mM IPTG. The choice of the Leu/Val precursor resulted in ^13^CH_3_ labeling of the pro-S methyl group in both amino acids in an otherwise per-deuterated ^12^C background.

### Blue Native PAGE analysis of OEP21 in chloroplasts

Chloroplasts were isolated as described in Stengel et al.^44^ and mixed with outer envelope vesicles in a ratio of 5:1. The mixture was centrifuged for 5 min at 6,000*g* at 4 °C, and the resulting pellet was resuspended in 750 mM aminocaproic acid, 50 mM Bis-Tris and 0.5 mM EDTA pH 7.0, supplemented with 1% DDM (dodecyl-β-d-maltoside, Roth). After a 10-min incubation on ice, the suspension was centrifuged for 10 min at 15,000*g* at 4 °C, and the supernatant was mixed with 10× loading dye (750 mM amino caproic acid, 5% Coomassie G-250). This was loaded onto a 7.5–15% acrylamide gel with 20 µg chlorophyll per lane. For subsequent western blotting, lanes were incubated in Towbin buffer (25 mM Tris pH 8.3, 192 mM glycine, 0.1% (w/v) SDS, 20% (v/v) methanol) with additional 0.9% (w/v) SDS and 50 mM DTT for 30 min before blotting.

### Proteolytic digest of outer envelope membranes and immunoprecipitation of OEP21

Isolated OEMs^21^, equivalent to 20 µg total protein per sample, were centrifuged at 256,000*g* for 10 min at 4 °C. The pellet was resuspended in 20 mM tricine pH 8.0 and 0.5 mM CaCl_2_ and treated with 200 ng trypsin from bovine pancreas (T1426, Sigma Aldrich) for 90 s at room temperature (RT). The digestion was stopped by addition of 1× cOmplete protease inhibitor (Roche), and proteins were solubilized by adding 1 volume of 2× SDS Laemmli loading buffer. Samples were run on 10% or 15% SDS gels. Immunoblotting was performed o PVDF membranes in Towbin buffer (25 mM Tris pH 8.3, 192 mM glycine, 1% SDS, 20% methanol) in a wet transfer apparatus for 1 h at RT at 300 mA or overnight at 4 °C at 60 mA. The membranes were blocked for at least 30 min with 1% skim milk in 50 mM Tris pH 7.6, 150 mM NaCl and 0.05% Tween 20 (TBS-T), and then were incubated with specific antibodies in 1:1,000 dilution in TBS-T for 2 h at room temperature. Subsequently, membranes were washed 3 times in TBS-T, then incubated with HRP-coupled secondary antibody (goat-anti-rabbit, Sigma Aldrich) in blocking buffer for 1 h at RT, and then were washed 3 times in TBS-T. Proteins were detected by chemiluminescence after incubating the membranes in 100 mM Tris pH 8.5, 25 mM luminol, 4 mM coumaric acid and 0.2% H_2_O_2_ for 1 min. Immunoprecipitation of trypsin-treated outer envelope membranes was performed with 1 mg total protein, digested as described above. Membranes were solubilized in 1% SDS, diluted 1:10 with 50 mM Tris pH 7.6, 150 mM NaCl, 1× cOmplete and incubated with OEP21 antiserum coupled to ProteinA Sepharose beads (GE Healthcare) for 1 h at RT. Beads were washed twice with 50 mM Tris pH 7.6, 150 mM NaCl, 0.1% SDS, 1× cOmplete and bound proteins were eluted by boiling in Laemmli loading buffer. Proteins were separated on a 12.5% Tricine gel, blotted onto PVDF as described above and subsequently stained with Coomassie Brilliant Blue (G). The upper band corresponding to the digestion fragment identified in the immunoblot was cut out and subjected to Edman sequencing (TopLab).

### Protein crosslinking

Protein crosslinking in detergent micelles was performed in 20 mM HEPES-KOH, pH 7.5, and 50 mM KCl, with a final OEP21 concentration of 20 µM in the presence of 0.1% of LDAO. A 50× molar excess of the amino-selective crosslinker BS^3^ (ThermoFisher Scientific, cat. no. 21580) was applied in the absence or presence of the respective metabolite at a 5 mM concentration for 30 min at room temperature, following 5 min of preincubation with the metabolites. The reaction was quenched by the addition of 50 mM Tris pH 7.5 for 15 min. For crosslinking in liposomes, liposomes were prepared as described in the next section in the absence or presence of 5 mM GAP or ATP using 10 μM OEP21. A 50× molar excess of BS^3^ was applied to liposomes for 30 min at RT. The reaction was quenched by addition of 50 mM Tris pH 7.5 for 15 min at RT. Finally, the samples were analyzed by SDS–PAGE. For crosslinking of cysteines by Cu^2+^, 20 µM of protein samples in 20 mM HEPES-KOH, pH 7.5, 50 mM KCl and 0.1% LDAO were incubated in the absence or presence of 1 mM CuSO_4_ for 15 min. Finally, the samples were analyzed by SDS–PAGE under non-reducing conditions. To detect the effect of cysteine oxidation on overall protein oligomerization, we applied Cu^2+^-crosslinking and then preincubated the samples in the absence or presence of 5 mM GAP or ATP for 5 min at RT. Finally, the BS^3^ crosslinking experiment was performed as described above, followed by analysis by SDS–PAGE.

### Preparation of liposomes and metabolite transport assays

Soybean polar lipid extract (Avanti Polar Lipids, 541602C) was first dried under a stream of nitrogen gas and solubilized at a concentration of 10 mg ml^–1^ in 20 mM HEPES-KOH pH 7.5, 250 mM KCl and 1% LDAO using an ultrasonic bath. After addition of 2 mM ATP on ice, OEP21 was added into the lipid–ATP mix. For every reconstitution experiment, a control liposome sample was prepared in parallel using the same lipid mix but an identical volume of SEC buffer (20 mM HEPES-KOH pH 7.5, 50 mM KCl, 0.5 mM EDTA, 5 mM DTT and 0.1% LDAO) was added instead of the purified protein. After shaking the sample for 30 min at 10 °C, liposomes were formed by gradual removal of the detergent from the mixture using 3 rounds (2 × 100 mg ml^–1^, 1 × 200 mg ml^–1^) of Bio-Beads SM2 resin (Bio-Rad) for 1.5 h at 4 °C. Following 3 freeze–thaw cycles, liposomes were passed through a 0.2-µm filter 15 times in a mini extruder (Avanti Polar Lipids).

For transport assays, the molecule to be tested (2 mM GAP, 2 mM ATP (already contained for each reconstitution except for the BS^3^ crosslinking experiment in the presence of only GAP), 200 µM MANT-ATP, 2 mM NADH, 140 µM bromophenol blue, 100 µM magnesium green (Thermo Fisher Scientific, M3733), 2 mM NADPH, 1.5 mM FAD, 30 µM Atto532 or 2.5 mM vitamin B_12_) was added into liposomes just before the 3 freeze–thaw cycles and extrusion through a 0.2-µm membrane. Subsequently, the liposome samples were applied to PD10 desalting columns equilibrated with 20 mM HEPES-NaOH pH 7.5 and 250 mM NaCl, and the liposome fractions were collected. The content of each molecule inside liposomes was quantified in the presence of 0.2% Triton X-100 to disintegrate the liposomes. The 846-Da monomeric ‘Atto532′ molecule was generated by incubating Atto532-maleimide (ATTO-TEC, AD532-41) with a twofold excess of β-mercaptoethanol, and the dimeric 1688 Da ‘2× Atto532′ molecule was generated by incubating DTT and Atto532-maleimide in a 1:2 molar ratio for at least 2 h at RT.

The GAP content was measured through NADH fluorescence (excitation, 340 nm; emission, 450 nm), which was generated by an enzyme-coupled NADH assay in the presence of 1 mM NAD^+^, 10 mM potassium phosphate pH 7.5, 0.02 mg ml^–1^ GAPDH. Excitation 340 nm and emission 450 nm were also used for detection of NADH and NADPH. ATP and MANT-ATP content of the liposomes were measured using an Invitrogen ATP determination kit (Thermo Fisher Scientific). Magnesium green, FAD and Atto532 contents were measured by fluorescence detection at an excitation of 506 nm and an emission of 546 nm, an excitation of 450 nm and an emission of 520 nm and an excitation of 532 nm and an emission of 572 nm, respectively. Bromophenol blue and vitamin B_12_ contents were measured by absorbance at 590 nm and 363 nm, respectively.

For the detection of GAP translocation in the presence of ATP or MgCl_2_, 5 mM GAP and 3 mM ATP (final 5 mM) and/or 10 mM MgCl_2_ were added just before the 3 freeze–thaw cycles, and the extrusion step. The PD10 SEC column runs were conducted in the presence or absence of 5 mM ATP and/or 10 mM MgCl_2_. For Δ*Ψ* measurements, 1.5 µl of liposomes in 20 mM HEPES-KOH pH 7.5, 250 mM KCl were diluted in 1.5 ml of 20 mM HEPES-NaOH pH 7.5, 250 mM NaCl containing 0.5 µM of potentiometric fluorescent dye DiSC_3_(5). Time course fluorescent measurements were done using spectrofluorometer FP-8300 (Jasco) at 622 nm excitation and 670 nm emission wavelengths. Then, 2 nM valinomycin and 250 mM KCl were added at the indicated time points for generation and dissipation of Δ*Ψ*, respectively. Data were plotted with OriginPro (OriginLab).

### Proteolytic cleavage of recombinant OEP21 in liposomes

Liposomes containing 10 µM OEP21 were prepared as described above in the absence of ATP. Trypsin was added to samples in the presence or in absence of 0.2% TX100, 5 mM GAP and/or 5 mM ATP. Sample aliquots were quenched by addition of 1 mM PMSF at the indicated time points and were analyzed by SDS–PAGE.

### Circular dichroism spectroscopy

CD spectra and thermal transitions were recorded on a Jasco J-715 spectropolarimeter (Jasco Deutschland). Far-UV CD spectra were recorded at 20 °C from 190 to 260 nm at a scanning speed of 50 nm min^–1^ and 5 accumulations in a 1 mm path-length cuvette. Melting temperatures were obtained by monitoring the CD signal at 215 nm during continuous heating from 20 to 100 °C, with a heating rate of 1 °C min^–1^. Curve fitting to a custom Boltzmann equation^45^ and plotting was done with the software ProFit 7 (QuantumSoft). All protein samples had a concentration of 10 µM in 10 mM Na-phosphate pH 7.0, 0.5 mM DTT and 0.1% LDAO.

### Isothermal titration calorimetry

ITC experiments were performed with a MicroCal PEAQ-ITC instrument (Malvern Panalytical) at 25 °C in 10 mM HEPES pH 7.0, 20 mM NaCl, 0.5 mM EDTA, 1 mM DTT, 50 mM LDAO. For titrations with the metabolites, the OEP21 concentration in the cell was 75 µM, whereas the concentration in the syringe was 1.5 mM for ATP, CTP, UTP, 2.5 mM for ADP and 5 mM for AMP. To determine the affinity of ATP, GAP or phosphate to magnesium, a buffer containing 10 mM HEPES pH 7.0 and 20 mM NaCl was used. Curve fitting and data analysis were done with the Malvern PEAQ-ITC software.

### Fluorescence polarization

FP assays were performed on a spectrofluorometer FP-8300 (Jasco) equipped with a water bath cooling system MCM-100 (Jasco). Samples were put in a 1-cm path-length quartz cuvette, and the FP of the fluorescently labeled ATP, 2′-(or-3′)-*O*-(*N*-methylanthraniloyl) adenosine 5′-triphosphate (MANT-ATP, Thermo Scientific) was measured at 25 °C in 10 mM HEPES pH 7.0, 20 mM NaCl, 0.5 mM EDTA, 1 mM DTT and 0.1% LDAO. A buffer containing no EDTA was used when MgCl_2_ was present. Excitation was done at 356 nm and the emission was detected at 448 nm with a 5-nm bandwidth. To determine the affinity of MANT-ATP for OPE21 variants, increasing concentrations of the protein were titrated stepwise to 200 nM MANT-ATP. Depending on the binding affinity, the final protein concentration was between 500 nM and 800 nM. *K*_D_ values were derived by fitting the data to a one-site binding model. Using the same workflow and adjusted final OEP21 concentrations, the affinities of OEP21-WT for MANT-ATP in presence of different concentrations of NaCl (0, 0.5, 1, 2 M) and MgCl_2_ (0, 1, 10, 50 mM) were determined. To determine IC_50_ values for the competitive binding of MANT-ATP and Na-phosphate, GAP or GAP + MgCl_2_ (2:1 concentration ratio), FP was measured of a preformed complex of 1 µM MANT-ATP and 1 µM OEP21-WT or OEP21ΔL5 in the presence of increasing concentrations of the competitor. In addition, the binding affinity for fluorescently labeled MANT-5′-guanylyl imidodiphosphate (GMPPNP, Thermo Scientific), was determined as described for MANT-ATP. All FP experiments were performed as triplicates.

### Nuclear magnetic resonance structure determination

NMR experiments were done at 308 K on Bruker AvanceIII spectrometers operating at 800, 900 or 950 MHz proton frequency with cryogenic probes and were controlled with Topspin 4.0 (Bruker Biospin). For backbone resonance assignments, a set of TROSY-type 3D-experiments was recorded^46^ as well as a 3D-^15^N-edited-[^1^H,^1^H]-NOESY-TROSY (200 ms mixing time) with a 400 µM *U*-^2^H,^13^C,^15^N-labeled OEP21 sample in 20 mM Na-phosphate, pH 6.0, 50 mM NaCl, 0.5 mM EDTA, 5 mM DTT, 5 mM ATP, 300 mM per-deuterated *d*_*31*_-LDAO (FB Reagents). All 3D-NOESY (types HNH, HCH, CCH; 200 ms mixing time) experiments for structure determination were run in a non-uniformly sampled (NUS) manner with 15–20% sampling density with a uniformly ^2^H,^15^N-labeled OEP21 sample containing selective methyl group labels (Ile-δ1, Leu-δ2, Val-γ2, Ala-β) as well as incorporated ^1^H,^15^N-labeled aromatic amino acids (Phe and Tyr). The NUS sampling schedule was obtained by the Poisson-gap method^47^. For rapid spectra reconstruction, we employed iterative soft thresholding (IST)^48^. All NUS-3D spectra were processed with NMRpipe^49^. All other spectra were processed with Topspin3.5 (Bruker Biospin). Resonance assignment and NMR data analysis were done with NMRFAM-Sparky^50^. Chemical-shift-based backbone dihedral angle restraints were calculated with the program TALOS+ (ref. ^51^). Structure calculation and refinement were performed with Xplor-NIH^52^ using standard protocols. Structural statistics (Table 1) are reported for the ten lowest-total-energy structures. Ramachandran analysis of backbone angles was done with PROCHECK-NMR^53^ using the best-energy structure (most favored regions: 81.3%; additionally allowed regions: 12.9%; generously allowed regions: 4.5%; disallowed regions: 1.3%). Calculation of the electrostatic potential of the OEP21 pore interior was done in PyMol (Schrödinger) and visualized with the program HOLLOW^54^. The pore diameter was analyzed with the program CHEXVIS^55^. A structural model of the dimeric, disulfide-bridged (through Cys109) form of OEP21 was calculated with Xplor-NIH using standard scripts using non-crystallographic symmetry restraints. The resulting structural model was subjected to a MD simulation of 100 ns duration at 303 K in a DMPC/DMPG lipid bilayer with the program NAMD^56^ on an in-house CPU cluster.

### Nuclear magnetic resonance titrations

Metabolite and detergent titrations were monitored by a series of 2D-[^15^N,^1^H]-TROSY experiments at 303 K with LDAO-solubilized 400 µM ^2^H,^15^N-labeled OEP21 in 20 mM HEPES pH 7.0, 50 mM NaCl, 0.5 mM EDTA, 5 mM DTT. Typically, 48 transients were recorded per increment, with 128 complex points in the indirect ^15^N dimension. Curve fitting of NMR binding isotherms was done with a full binding model accounting for the relatively high protein concentration (>*K*_D_) required for NMR experiments^57^. Chemical shift perturbations in the ^1^H and^15^N spectral dimensions were scaled using the distribution of nucleus-specific chemical shift changes in proteins^58^.

Paramagnetic relaxation enhancements with the spin-labeled fatty acid 16-doxyl-stearic acid (16-DSA) were monitored with 2D-[^15^N,^1^H]-TROSY experiments at 308 K using a recycle delay of 2.5 s. Two experiments were recorded with ^2^H,^15^N-labeled OEP21 in 20 mM Na-phosphate pH 6.0, 50 mM NaCl, 0.5 mM EDTA, 5 mM DTT, 150 mM LDAO with and without 2 mM 16-DSA. For visualization, peak intensity ratios (±2 mM 16-DSA) were plotted against the residue number.

### Amide hydrogen exchange nuclear magnetic resonance experiments

Amide hydrogen exchange was quantified with CLEANEX NMR experiments^59^ using different mixing times (2–100 ms) at 303 K. Fitting of the built-up curves was done with a mono-exponential equation. In addition, hydrogen exchange was qualitatively monitored with 3D ^15^N-edited-[^1^H,^1^H]-NOESY-TROSY experiments with a mixing time of 40 ms. The occurrence of sequential amide-amide cross peaks as well as the hydrogen exchange with water was analyzed. These experiments were recorded with 400 µM ^2^H,^15^N-labeled OEP21 in the apo form at a high LDAO concentration (300 mM), as well as in presence of 2 mM ATP or 5 mM GAP in 20 mM Na-phosphate pH 6.0, 50 mM NaCl, 0.5 mM EDTA and 5 mM DTT.

### Molecular dynamics simulations

OEP21 was embedded in a bilayer consisting of 202 DMPC molecules and solvated in an aqueous 0.15 M KCl solution, using the membrane builder of the CHARMM-GUI web-server^60–62^. The ligands GAP^2−^ and ATP^4−^ were placed outside the pore. All simulations (duration of 2–4 µs) were performed using the GPU accelerated CUDA version of PMEMD^63^, part of the AMBER18 package^64^ (see also Supplementary Methods). The unbiased simulations were carried out at a pressure of 1 bar. Simulations with an exterior electric field of 5.625 mV A^–1^ were performed without pressure coupling (NVT ensemble). The target temperature for all simulations was 303 K.

### Reporting summary

Further information on research design is available in the Nature Portfolio Reporting Summary linked to this article.

## Online content

Any methods, additional references, Nature Portfolio reporting summaries, source data, extended data, supplementary information, acknowledgements, peer review information; details of author contributions and competing interests; and statements of data and code availability are available at https://doi.org/10.1038/s41594-023-00984-y.

## Supplementary information

Supplementary Information Supplementary methods for MD simulations

Reporting Summary

Supplementary Data 1 Disulfide-linked model of the OEP21 dimer

Supplementary Data 2 Stereo-image of OEP21 backbone structure

Supplementary Video 1 MD simulation for Fig. 4a

## Source data

Source Data Fig. 1 Source data for Fig. 1

Source Data Fig. 2 Source data for Fig. 2

Source Data Fig. 2 Uncropped blots and gels for Fig. 2

Source Data Fig. 3 Source data for Fig. 3

Source Data Fig. 4 Source data for Fig. 4

Source Data Extended Data Fig. 2 Uncropped blots and gels for Extended Data Fig. 2

Source Data Extended Data Fig. 3 Source data for Extended Data Fig. 3

Source Data Extended Data Fig. 4 Source data for Extended Data Fig. 4

Source Data Extended Data Fig. 5 Source data for Extended Data Fig. 5

Source Data Extended Data Fig. 7 Source data for Extended Data Fig. 7

Source Data Extended Data Fig. 10 Source data for Extended Data Fig. 10

## Data Availability

The NMR chemical shift information and the structural coordinates of OEP21 have been deposited at the Biological Magnetic Resonance Data Bank (accession code 34589) and RCSB (accession code 7BGH) data banks. The coordinates of the structural model of dimeric, disulfide-bridged OEP21 can be accessed from Figshare (https://doi.org/10.6084/m9.figshare.22434565.v1). Stereo image of the OEP21 structure is available with the article (Supplementary Data 1). The MD simulation trajectory of GAP translocation across OEP21 is provided as Supplementary Video 1. Source data are provided with this paper.
