# Supplementary material for: Structural basis of metabolite transport by the chloroplast outer envelope channel OEP21
Source: Nat Struct Mol Biol. 2023 May 8;30(6):761–9. doi: 10.1038/s41594-023-00984-y (PMC10279527; doi:10.1038/s41594-023-00984-y)
Supplement: Supplementary file 1 — Supplementary methods for MD simulations [file 41594_2023_984_MOESM1_ESM.pdf]

nature structural & molecular biology

Article https://doi.org/10.1038/s41594-023-00984-y

Structural basis of metabolite transport by the chloroplast outer envelope channel OEP21

In the format provided by the authors and unedited

Supplementary methods

Molecular Dynamics (MD) Simulations

The MD simulations have been performed using the CUDA version of PMEMD [1], which is part of the AMBER18 simulation package[2]. Amino acids, water molecules and lipids were described by the AMBER14SB [3], TIP3P [4] and LIPID17 (only LIPID14 has been published [5]) force fields, respectively. The parameters for ATP were taken from the AMBER parameter database of the University of Manchester [6]. GAP has been parametrized using the Antechamber module of AMBER18, utilizing the GAFF2 force field [7]. The RESP [8] partial charges (Hartree-Fock at 6-31G* level) have been calculated from a structure that was optimized to a gas-phase energy minimum, at the B3LYP/TZVP – level. All QM calulculations have been performed with GAUSSIAN09[9]. The cut-off for all pair-wise interactions was set to 9 Å in the simulations. Long-range Coulomb interactions were accounted for by the particle mesh ewald method [10], while long-range van-der-Waals effects were estimated by a dispersion correction model. Periodic boundary conditions were used and the dimension of the simulation boxes was approx. 88 Å by 88 Å by 104 Å. The simulations were performed at 303.15 K, using the Langevin thermostat [11] with a collision frequency of 1 ps^{-1} . The NPT ensembles were sampled at an ambient pressure of 1.0 bar, employing the Berendsen barostat [12] with a relaxation time of 1 ps. In the production runs, time steps of 4.0 fs were used, which was enabled by utilizing both, the SHAKE algorithm [13] and hydrogen mass repartitioning [14].

Simulation System Setup

The starting structures have been generated by embedding OEP21 in a DMPC bilayer (202 molecules), solvated by 17255 water molecules and 0.15 M KCl. This was achieved by using the bilayer builder of the CHARMM-GUI server [15-17]. For all tritatable amino acids, the standard protonation states were chosen (charged GLU, ASP, ARG, LYS and HIS protonated at the ε-nitrogen). Subsequently, 13 different starting structures have been generated by adding one ATP or GAP molecule to the simulation box in random positions. For ATP, 8 different starting structures were generated (5 times ATP was positioned closer to the cytoplasmic entrance of OEP21 and three starting structures featured ATP in closer proximity to the IMS interface). The 5 GAP starting structures featured the metabolite closer to the IMS entrance in two cases, while in the other three simulations the intial position of GAP was in the cytosol. In all cases, the metabolites have been positioned in a way that they were initially not in contact with the protein but completely solvated. Due to the periodic boundary conditions applied to the system, the IMS and cytosolic sites from two adjacent boxes are in contact with each other, thus allowing the metabolites to venture from the IMS to the cytosol (and *vice versa*) not only by passing through OEP21, but also by leaving one box and entering the neighboring one (while always remaining in the aqueous environment). This switching between IMS and cytosole happend numerous times in many of the simulations, until the metabolite was finally captured by charged residues located at one of the two openings of OEP21.

Simulation Protocol

Prior to the 2 µs sampling phase, a 7-step minimization and equilibration protocol was applied to each starting structure (the standard equilibration protocol, established by CHARMM-GUI).

Details on this protocol can be found on table S1.

	Steps	Time Step	K Protein	K Lipid	K Dihed.	Temperature	Pressure
**min**	2000	minimization	10.0	2.5	250	---	---
**Eq1**	125000	1fs	10.0	2.5	100	303.15K	NVT
**Eq2**	125000	1fs	5.0	2.5	50	303.15K	NVT
**Eq3**	125000	1fs	2.5	1.0	50	303.15K	1 bar
**Eq4**	250000	2fs	1.0	0.5	50	303.15K	1bar
**Eq5**	250000	2fs	0.5	0.1	25	303.15K	1bar
**Eq6**	250000	2fs	0.1	0.0	0	303.15K	1bar

Table S1: *Overview over the 7 equilibration steps performed for all simulations. The initial minimization is followed by six equilibration simulations with decreasing force constants (K) on positional restraints of amino acids (Protein, all atoms), positional restraints on lipid headgroups (Lipid, phosphorus atom) as well as restraints on the lipid dihedrals (Dihed.).*

Supplementary Information Data

Molecular Dynamics Simulations

We assessed the binding stabilities of simulation end-states by performing Molecular Mechanics Poisson Boltzmann Surface Area (MMPBSA) calculations on the last 200 or 400 ns (depending on the longevity of the binding modes) of each simulation. We used the MMPBSA.py script[18] provided with the AMBER18 package. Since the Poisson Boltzmann (PB) routine of AMBER18 was unable to recognize one of the atom types in the ATP force field, the PB calculations were performed with flags inp=1 and radiopt=0. This resulted in slightly different surface area (SA) terms compared to the standard method in AMBER18 (they are more similar to the ones used in AMBER Generalized Born calculations). In order to ensure comparability of the results, the same method was also used for MMPBSA calculations of the GAP binding modes.

The free energy calculations have been performed with the same salt concentration as the simulations (150 mM). We did not perform estimations on entropy change, therefore the resulting free energies cannot be directly compared to experimental ΔG values. However, since we compared very similar systems, the resulting affinity estimates allow for comparison between the different binding modes. The results of free energy calculations can be found in **Tab. S2** (ATP) and **Tab. S3** (GAP). Snapshots of the end states of each simulation can be found in **Fig. S4**.

Simulation	ΔG [kcal/mol]	Std. Error	From	Binding Region
ATP 1	-115.97	2.07	Cytoplasm	Loop (cytopl.)
ATP 2	-45.65	0.90	Cytoplasm	Loop (cytopl.)
ATP 3	-56.96	0.54	Cytoplasm	Loop (cytopl.)
ATP 4	-52.26	1.34	Cytoplasm	Loop (cytopl.)
ATP 5	-68.10	0.66	Cytoplasm	Loop (cytopl.)
ATP 6	-84.87	1.49	IMS	Barrel
ATP 7	-88.56	1.10	IMS	Barrel
ATP 8	-88.94	0.94	IMS	Barrel
Average	-75.16	1.10	---	---

Table S2: *Results of MMPBSA calculations of the ATP simulations. Snapshots depicticting the corresponding binding mode can be found in Extended Data Fig. 5. Column "From" indicates the direction of substrate binding, while "Binding Region" highlights the location of the studied (and final) binding event.*

Simulation	ΔG [kcal/mol]	Std. Error	From	Binding Region
GAP 1	-33.13	0.55	Cytoplasm	Loop (cytopl.)
GAP 2	-37.99	0.67	IMS	Barrel
GAP 3	-36.24	0.54	Cytoplasm	Barrel
GAP 4	-65.53	0.55	IMS	Barrel
GAP 5	-56.71	0.85	IMS	Barrel
Average	-45.92	0.63	---	---

Table S3: *Results of MMPBSA calculations of the GAP simulations. Snapshots depicticting the corresponding binding mode can be found in Extended Data Fig. 5. Column "From" indicates the direction of substrate binding, while "Binding Region" highlights the location of the studied (and final) binding event.*

The free energy calculations predict differences in complex stabilities between GAP and ATP binding events: ATP binding is on average 29.24 kcal/mol more stable than GAP binding, which serves as a possible explanation for why GAP can easily pass through the channel whereas ATP seems to block it rather than being transported to the other side of the membrane. Tables S4 and S5 show all contributions to the total free energy of binding for both systems. By comparing the ATP to the GAP averages, we see that the stronger electrostatic interactions of ATP, including counteracting Poisson Boltzmann energy changes (to account for the higher desolvation penalty associated with an increase of ionic contacts, we have to sum electrostatic and PB energies) account for roughly 50% of the affinity difference between GAP and ATP (14.74 kcal/mol). This is mainly due to the higher charge

of ATP and it being able to form more salt bridges with the basic residues of OEP21. The other 50% of the stability difference (14.13 kcal/mol) between the systems, is due to stronger van der Waals interactions between OEP21 and ATP. This is likely caused by the adenine moiety which is not only very large but is also able to form stacking interactions with certain OEP21 residues (e.g. Arg, Phe, Tyr). Columns 4 and 5 (“From” and “Binding Region”) in tables S2 and S3 indicate from which side OEP21 got approached by the substrate (column 4) and where it actually ended up at the end of the simulation (column 5). In case of GAP, no matter from where it came from, the substrate ended up binding completely in the channel’s barrel region in all but one of the simulations: In the GAP 1 simulation the substrate is still partially attached to one of the cytoplasmic loops.

ATP on the other hand binds in the barrel only when it enters from the IMS. If ATP is approaching from the cytoplasmic side, it was always trapped by the long and flexible cytoplasmic loop (L5). In contrast to the GAP 1 simulation where the substrate theoretically could be transported deeper into the channel without restarting the entire binding process, ATP always stayed in the bound state on the outer surface of the channel protein. From these binding sites, ATP could theoretically only enter the barrel region after completely dissociating from the protein and binding somewhere else.

The simulation results therefore suggest that these loop regions serve a gatekeeping purpose and prevent especially larger molecules such as ATP, from trasnlocating from the cytoplasm to the IMS. Since we were able to sample three barrel binding events and five to the loop, we can compare mean binding affinities of ATP to the loop and to the inside of the β-barrel. By forming averages of free binding energies of simulations ATP 1 to ATP 5 and ATP 6 to ATP 8, respectively, we find that on average the affinity of ATP to the barrel is 19.67 kcal/mol higher than to the loop. If we consider simulation ATP 1 to be an outlier due to the uncharacteristically high affinity and remove it from the data set, we get an even higher energy difference of 31.72 kcal/mol. In any case, the data suggests that ATP binds tightly into the OEP21 channel, while binding events to the cytoplasmic loop can be expected to be more short lived. The reason for the energy differences between loop and barrel binding can again be taken from the energy decomposition (**Tab. S4**). If we sum over the electrostatic binding contribution (EEL) and the Poisson Boltzmann energy differences (EPB, the difference in solvation energy between bound and unbound states) for ATP 1 to ATP 5 and do the same for the β-barrel binding events (ATP 6, 7, 8), we find that the improved electrostatics of the barrel binding events provide 18.63 kcal/mol more stabilization over binding events sampled in the loop region. This accounts for approximately 95% of the overall difference in free energy.

For the GAP simulations, even though there is only one (partial) loop binding event, we also compared the energy contributions and find that complete β-barrel binding events exhibit complex stabilities which are on average increased by 15.99 kcal/mol. Similar to ATP, electrostatics and desolvation (EPB) account for the majority of the overall difference: 21.08 kcal/mol (which is mainly

counteracted by slightly less favorable van der Waals interactions in the barrel binding case). Thus, based on these calculations, the OEP21 channel is able to selectively attract metabolites into the β-barrel interior for further transport (in the case of GAP) or stable binding and channel inhibiton (with ATP).

**Simulation**	**Electrostatic (EEL)**	**EPB**	**EEL+EPB**	**VdW**	**SA**
ATP 1	-1520.43	1430.66	-89.77	-22.38	-3.82
ATP 2	-1158.23	1143.47	-14.76	-26.93	-3.96
ATP 3	-1226.78	1189.80	-36.98	-16.62	-3.36
ATP 4	-1061.67	1025.87	-35.80	-13.78	-2.68
ATP 5	-1192.59	1142.53	-50.06	-15.23	-3.36
ATP 6	-1551.58	1487.89	-63.69	-17.59	-2.68
ATP 7	-1513.81	1446.66	-67.14	-17.78	-2.81
ATP 8	-1706.56	1645.10	-61.46	-22.98	-3.68
Average	-1405.24	1349.61	-55.63	-17.47	-3.02

Table S4: *Energy contributions of the ATP MMPBSA calculations. All values are given as energy differences between bound and unbound states in kcal/mol.*

**Simulation**	**Electrostatic (EEL)**	**EPB**	**EEL+EPB**	**VdW**	**SA**
GAP 1	-629.71	605.68	-24.03	-7.19	-1.91
GAP 2	-736.25	700.17	-36.09	-0.38	-1.53
GAP 3	-718.98	687.02	-31.96	-2.64	-1.64
GAP 4	-631.76	568.01	-63.75	-0.36	-1.42
GAP 5	-771.23	722.59	-48.63	-6.13	-1.94
Average	-697.59	656.69	-40.89	-3.34	-1.69

Table S5: *Energy contributions of the GAP MMPBSA calculations. All values are given as energy differences between bound and unbound states in kcal/mol.*

Supplementary Method References

1 Salomon-Ferrer, R., Gotz, A. W., Poole, D., Le Grand, S. & Walker, R. C. Routine Microsecond Molecular Dynamics Simulations with AMBER on GPUs. 2. Explicit Solvent Particle Mesh Ewald. *J Chem Theory Comput* **9**, 3878-3888, doi:10.1021/ct400314y (2013).

2 AMBER 2018 (University of California, San Francisco, 2018).

3 Maier, J. A. *et al.* ff14SB: Improving the Accuracy of Protein Side Chain and Backbone Parameters from ff99SB. *J Chem Theory Comput* **11**, 3696-3713, doi:10.1021/acs.jctc.5b00255 (2015).
4 Jorgensen, W. L., Chandrasekhar, J., Madura, J. D., Impey, R. W. & Klein, M. L. Comparison of Simple Potential Functions for Simulating Liquid Water. *J Chem Phys* **79**, 926-935, doi:Doi 10.1063/1.445869 (1983).
5 Dickson, C. J. *et al.* Lipid14: The Amber Lipid Force Field. *J Chem Theory Comput* **10**, 865-879, doi:10.1021/ct4010307 (2014).
6 Meagher, K. L., Redman, L. T. & Carlson, H. A. Development of polyphosphate parameters for use with the AMBER force field. *J Comput Chem* **24**, 1016-1025, doi:10.1002/jcc.10262 (2003).
7 Wang, J., Wolf, R. M., Caldwell, J. W., Kollman, P. A. & Case, D. A. Development and testing of a general amber force field. *J Comput Chem* **25**, 1157-1174, doi:10.1002/jcc.20035 (2004).
8 Fox, T. & Kollman, P. A. Application of the RESP methodology in the parametrization of organic solvents. *J Phys Chem B* **102**, 8070-8079, doi:DOI 10.1021/jp9717655 (1998).
9 Gaussian 09, Revision A.02 (Gaussian, Inc., Wallingford CT, 2016).
10 Darden, T., York, D. & Pedersen, L. Particle Mesh Ewald - an N.Log(N) Method for Ewald Sums in Large Systems. *J Chem Phys* **98**, 10089-10092, doi:Doi 10.1063/1.464397 (1993).
11 Goga, N., Rzepiela, A. J., de Vries, A. H., Marrink, S. J. & Berendsen, H. J. Efficient Algorithms for Langevin and DPD Dynamics. *J Chem Theory Comput* **8**, 3637-3649, doi:10.1021/ct3000876 (2012).
12 Berendsen, H. J. C., Postma, J. P. M., Vangunsteren, W. F., Dinola, A. & Haak, J. R. Molecular-Dynamics with Coupling to an External Bath. *J Chem Phys* **81**, 3684-3690, doi:Doi 10.1063/1.448118 (1984).
13 Andersen, H. C. Rattle - a Velocity Version of the Shake Algorithm for Molecular-Dynamics Calculations. *J Comput Phys* **52**, 24-34, doi:Doi 10.1016/0021-9991(83)90014-1 (1983).
14 Hopkins, C. W., Le Grand, S., Walker, R. C. & Roitberg, A. E. Long-Time-Step Molecular Dynamics through Hydrogen Mass Repartitioning. *J Chem Theory Comput* **11**, 1864-1874, doi:10.1021/ct5010406 (2015).
15 Jo, S., Kim, T., Iyer, V. G. & Im, W. CHARMM-GUI: a web-based graphical user interface for CHARMM. *J Comput Chem* **29**, 1859-1865, doi:10.1002/jcc.20945 (2008).
16 Wu, E. L. *et al.* CHARMM-GUI Membrane Builder toward realistic biological membrane simulations. *J Comput Chem* **35**, 1997-2004, doi:10.1002/jcc.23702 (2014).
17 Lee, J. *et al.* CHARMM-GUI supports the Amber force fields. *J Chem Phys* **153**, 035103, doi:10.1063/5.0012280 (2020).
18 Miller, B. R., 3rd *et al.* MMPBSA.py: An Efficient Program for End-State Free Energy Calculations. *J Chem Theory Comput* **8**, 3314-3321, doi:10.1021/ct300418h (2012).
