# Supplementary material for: Structural basis of metabolite transport by the chloroplast outer envelope channel OEP21
Source: Nat Struct Mol Biol. 2023 May 8;30(6):761–9. doi: 10.1038/s41594-023-00984-y (PMC10279527; doi:10.1038/s41594-023-00984-y)
Supplement: Supplementary file 2 — Reporting Summary [file 41594_2023_984_MOESM2_ESM.pdf]

nature portfolio

Corresponding author(s): Franz Hagn
Last updated by author(s): Mar 27, 2023

Reporting Summary

Nature Portfolio wishes to improve the reproducibility of the work that we publish. This form provides structure for consistency and transparency in reporting. For further information on Nature Portfolio policies, see our Editorial Policies and the Editorial Policy Checklist.

Statistics

For all statistical analyses, confirm that the following items are present in the figure legend, table legend, main text, or Methods section.

n/a	Confirmed	
☐	☒	The exact sample size (*n*) for each experimental group/condition, given as a discrete number and unit of measurement
☐	☒	A statement on whether measurements were taken from distinct samples or whether the same sample was measured repeatedly
☒	☐	The statistical test(s) used AND whether they are one- or two-sided<br>*Only common tests should be described solely by name; describe more complex techniques in the Methods section.*
☒	☐	A description of all covariates tested
☒	☐	A description of any assumptions or corrections, such as tests of normality and adjustment for multiple comparisons
☐	☒	A full description of the statistical parameters including central tendency (e.g. means) or other basic estimates (e.g. regression coefficient) AND variation (e.g. standard deviation) or associated estimates of uncertainty (e.g. confidence intervals)
☒	☐	For null hypothesis testing, the test statistic (e.g. *F*, *t*, *r*) with confidence intervals, effect sizes, degrees of freedom and *P* value noted<br>*Give P values as exact values whenever suitable.*
☒	☐	For Bayesian analysis, information on the choice of priors and Markov chain Monte Carlo settings
☒	☐	For hierarchical and complex designs, identification of the appropriate level for tests and full reporting of outcomes
☒	☐	Estimates of effect sizes (e.g. Cohen's *d*, Pearson's *r*), indicating how they were calculated

Our web collection on statistics for biologists contains articles on many of the points above.

Software and code

Policy information about availability of computer code

Data collection	Topspin 4.0 (Bruker Biospin), SoftMaxPro 7.1 (Molecular Devices), Spectra Manager (Jasco Co), MicroCal PEAQ-ITC control software 1.41 (Malvern Panalytical)
Data analysis	Topspin 4.0 (Bruker Biospin), NMR Pipe, mac11_64 (NIH), NMRFAM Sparky (Univ of Wisconsin), Talos+ (NIH), Xplor-NIH 3.5 (NIH), Spectra Manager (Jasco Co), OriginPro (OriginLab), ProFit 7 (QuantumSoft), MacPyMol 1.6 (Schrödinger LLC), Chimera 1.14 (UCSF), HOLLOW (http://hollow.sourceforge.net), ChExVis (http://vgl.serc.iisc.ernet.in/chexvis/), AMBER18, CHARMM-GUI (Lehigh Univ), NAMD 2.14 (University of Illinois Urbana-Champaign), MicroCal PEAQ-ITC analysis software (Malvern), hmsIST v211 (Harvard Medical School), PROCHECK-NMR 3.5.4 (EMBL-EBI), SHAKE algorithm, GAUSSIAN09

For manuscripts utilizing custom algorithms or software that are central to the research but not yet described in published literature, software must be made available to editors and reviewers. We strongly encourage code deposition in a community repository (e.g. GitHub). See the Nature Portfolio guidelines for submitting code & software for further information.

Data

Policy information about availability of data

All manuscripts must include a data availability statement. This statement should provide the following information, where applicable:

- Accession codes, unique identifiers, or web links for publicly available datasets
- A description of any restrictions on data availability
- For clinical datasets or third party data, please ensure that the statement adheres to our policy

The NMR chemical shift information and the structural coordinates of OEP21 have been deposited at the BMRB (accession code 34589) and RCSB (accession code 7BGH) data banks, respectively. The coordinates of the structural model of dimeric, disulfide bridged OEP21 is contained in the supporting information data (Supplementary Data 1), as well as a stereo image of the OEP21 structure (Supplementary Data 2). The MD simulation trajectory of GAP translocation across OEP21 is provided as Supplementary Video 1. Where applicable, source data are provided for the main and extended data figures.

Human research participants

Policy information about studies involving human research participants and Sex and Gender in Research.

Reporting on sex and gender	n/a
Population characteristics	n/a
Recruitment	n/a
Ethics oversight	n/a

Note that full information on the approval of the study protocol must also be provided in the manuscript.

Field-specific reporting

Please select the one below that is the best fit for your research. If you are not sure, read the appropriate sections before making your selection.

☒ Life sciences ☐ Behavioural & social sciences ☐ Ecological, evolutionary & environmental sciences

For a reference copy of the document with all sections, see nature.com/documents/nr-reporting-summary-flat.pdf

Life sciences study design

All studies must disclose on these points even when the disclosure is negative.

Sample size	For the in vitro experiments the scattering of data was very low. Sample size was chosen as an uneven number, at least 3.
Data exclusions	clear outliers were excluded
Replication	For all assay data, 3 to 5 independent experiments were conducted (technical replicates). All attempts were successful but outliers were excluded.
Randomization	Not relevant. Samples at identical conditions were measured to obtain mean values and standard deviations.
Blinding	Not relevant. All data values at identical sample conditions were used for the analysis.

Reporting for specific materials, systems and methods

We require information from authors about some types of materials, experimental systems and methods used in many studies. Here, indicate whether each material, system or method listed is relevant to your study. If you are not sure if a list item applies to your research, read the appropriate section before selecting a response.

Materials & experimental systems

n/a	Involved in the study	
☐	☒	Antibodies
☒	☐	Eukaryotic cell lines
☒	☐	Palaeontology and archaeology
☒	☐	Animals and other organisms
☒	☐	Clinical data
☒	☐	Dual use research of concern

Methods

n/a	Involved in the study	
☒	☐	ChIP-seq
☒	☐	Flow cytometry
☒	☐	MRI-based neuroimaging

Antibodies

Antibodies used

goat-anti-rabbit HRP-coupled (Sigma Aldrich); anti-OEP21; anti-Toc75; anti-Toc64 (produced in house, see references). All specific antibodies were used at a 1:1000 dilution.

Validation

Non-commercial antibodies were produced in-house and validated in previous studies:

anti-OEP21: Bölter, B., Soll, J., Hill, K., Hemmler, R. & Wagner, R. A rectifying ATP-regulated solute channel in the
469 chloroplastic outer envelope from pea. EMBO J 18, 5505-5516, doi:10.1093/emboj/18.20.5505 (1999)

anti-Toc64: Sohrt, K. & Soll, J. Toc64, a new component of the protein translocon of chloroplasts. J Cell Biol 148,
491 1213-1221, doi:10.1083/jcb.148.6.1213 (2000)

anti-Toc75: Seedorf, M. & Soll, J. Copper chloride, an inhibitor of protein import into chloroplasts. FEBS Lett 367,
489 19-22, doi:10.1016/0014-5793(95)00529-i (1995)
