# Supplementary figures and images for: Structural basis of metabolite transport by the chloroplast outer envelope channel OEP21
Source: Nat Struct Mol Biol. 2023 May 8;30(6):761–9. doi: 10.1038/s41594-023-00984-y (PMC10279527; doi:10.1038/s41594-023-00984-y)

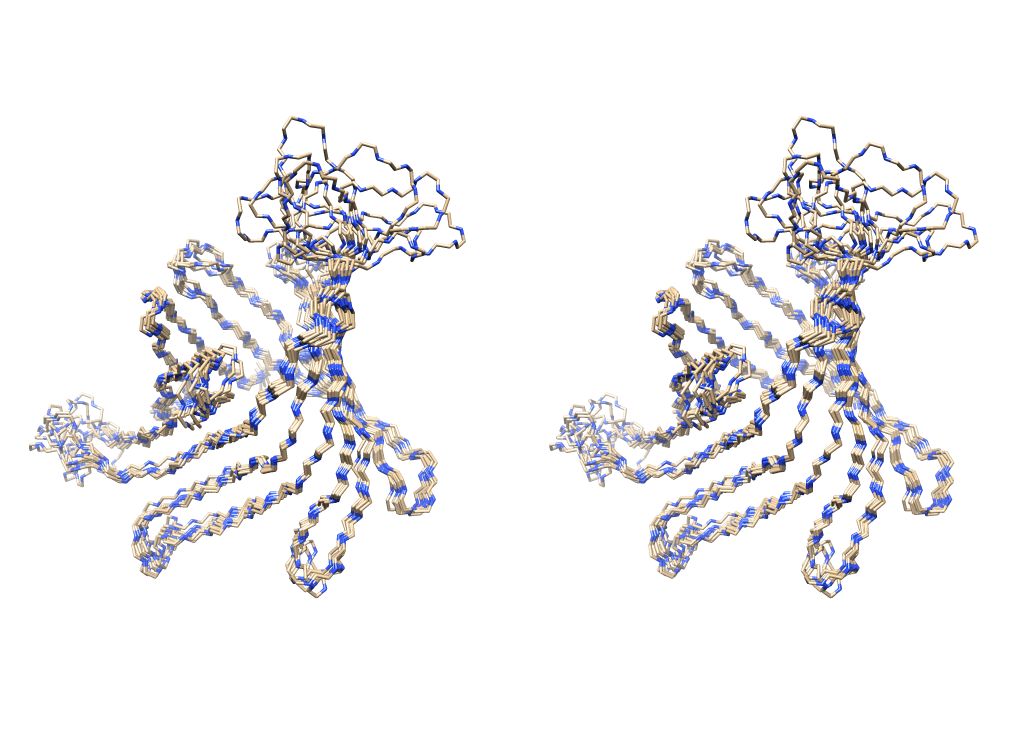

Supplement: Supplementary file 4 — Stereo-image of OEP21 backbone structure [file 41594_2023_984_MOESM4_ESM.png]

Figure 2a

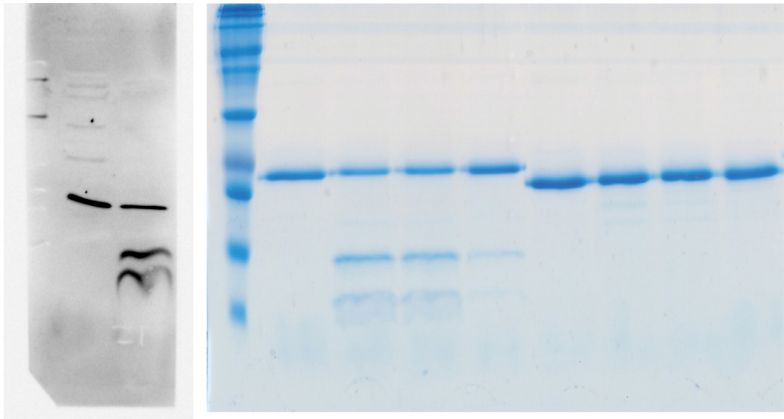

Figure 2b

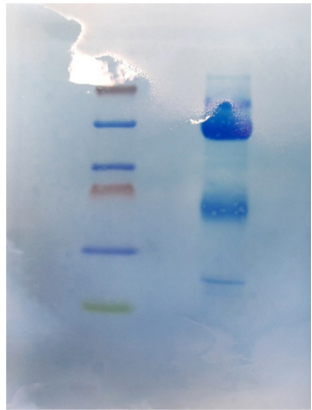

Figure 2d

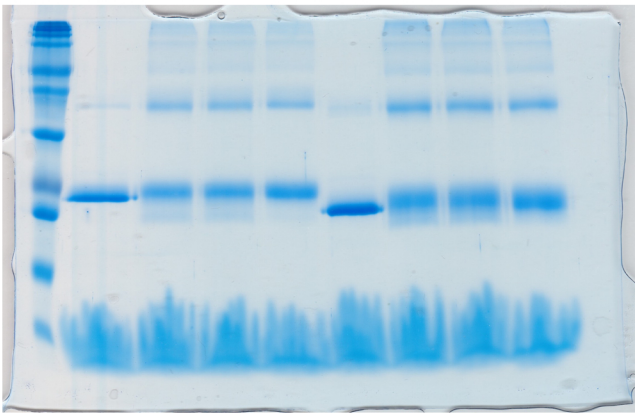

Figure 2g

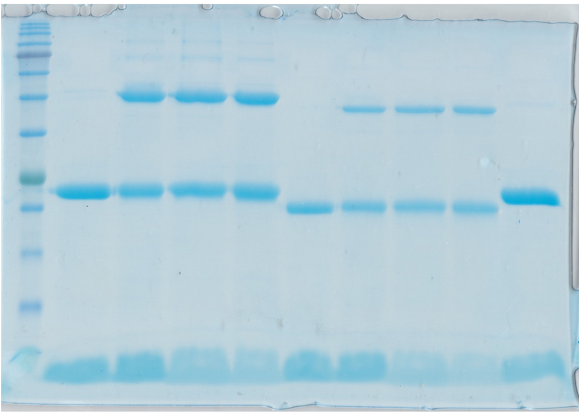

Supplement: Source Data Fig. 2 — Uncropped blots and gels for Fig. 2 [file 41594_2023_984_MOESM8_ESM.pdf]
