# Supplementary material for: Structural basis of metabolite transport by the chloroplast outer envelope channel OEP21
Source: Nat Struct Mol Biol. 2023 May 8;30(6):761–9. doi: 10.1038/s41594-023-00984-y (PMC10279527; doi:10.1038/s41594-023-00984-y)

ED. Fig. 2a

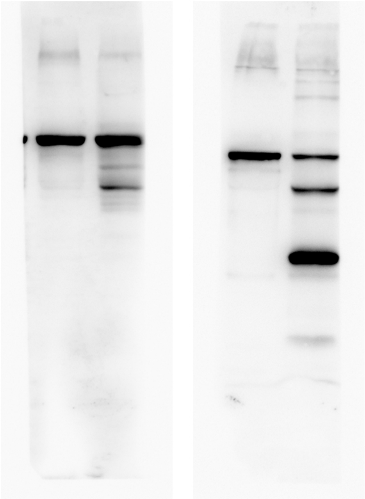

ED. Fig. 2b (Check uncropped Fig2b for Marker positions)

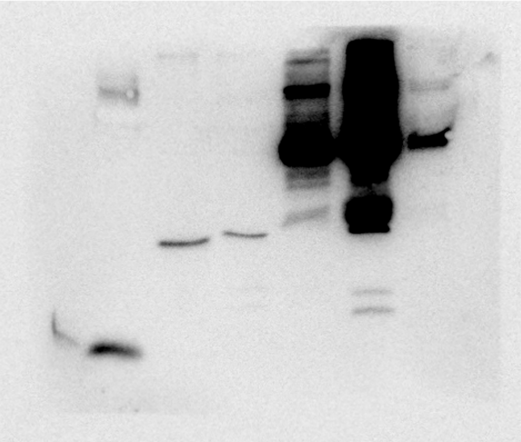

ED. Fig. 2e-f

OEP21

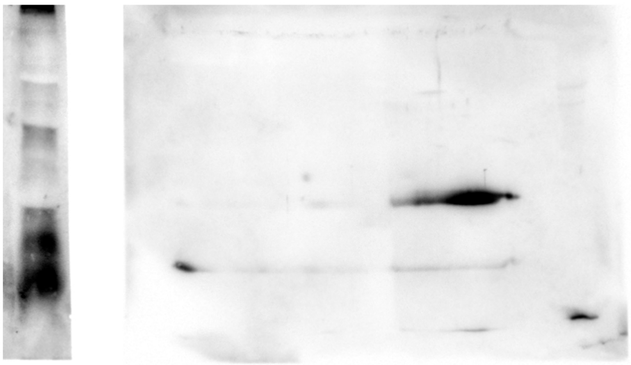

Tic110

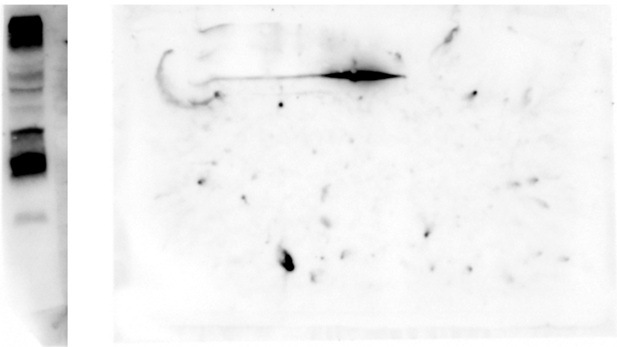

TOC34

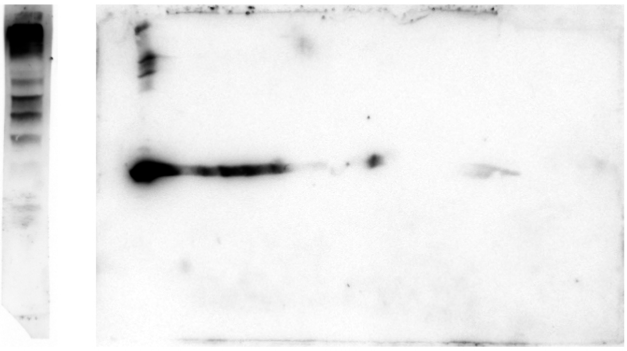

Chloroplast strips before decoration with specific antibodies

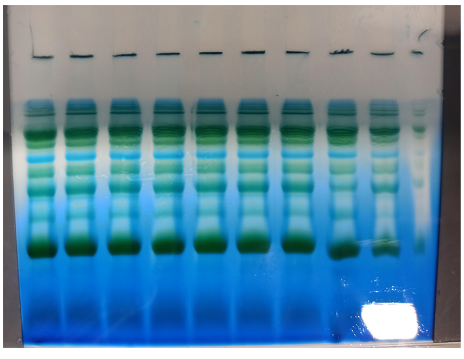

Guide to transfer marker bands to chloroplast lanes

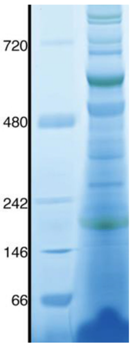

A replicate of OEP21 in 2nd SDS-PAGE dimension (not used for the manuscript)

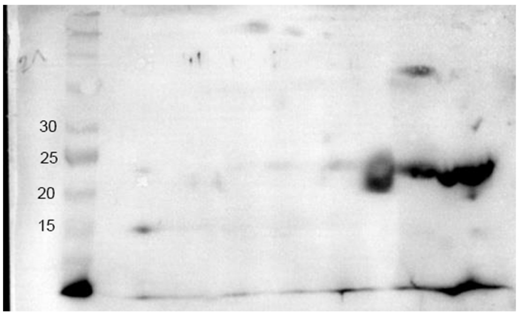

Supplement: Source Data Extended Data Fig. 2 — Uncropped blots and gels for Extended Data Fig. 2 [file 41594_2023_984_MOESM11_ESM.pdf]
